# Supplementary figures and images for: Phenotypic and Genomic Diversification in Complex Carbohydrate-Degrading Human Gut Bacteria
Source: mSystems. 2022 Feb 15;7(1):e00947-21. doi: 10.1128/msystems.00947-21 (PMC8845570; doi:10.1128/msystems.00947-21)

Figure S1

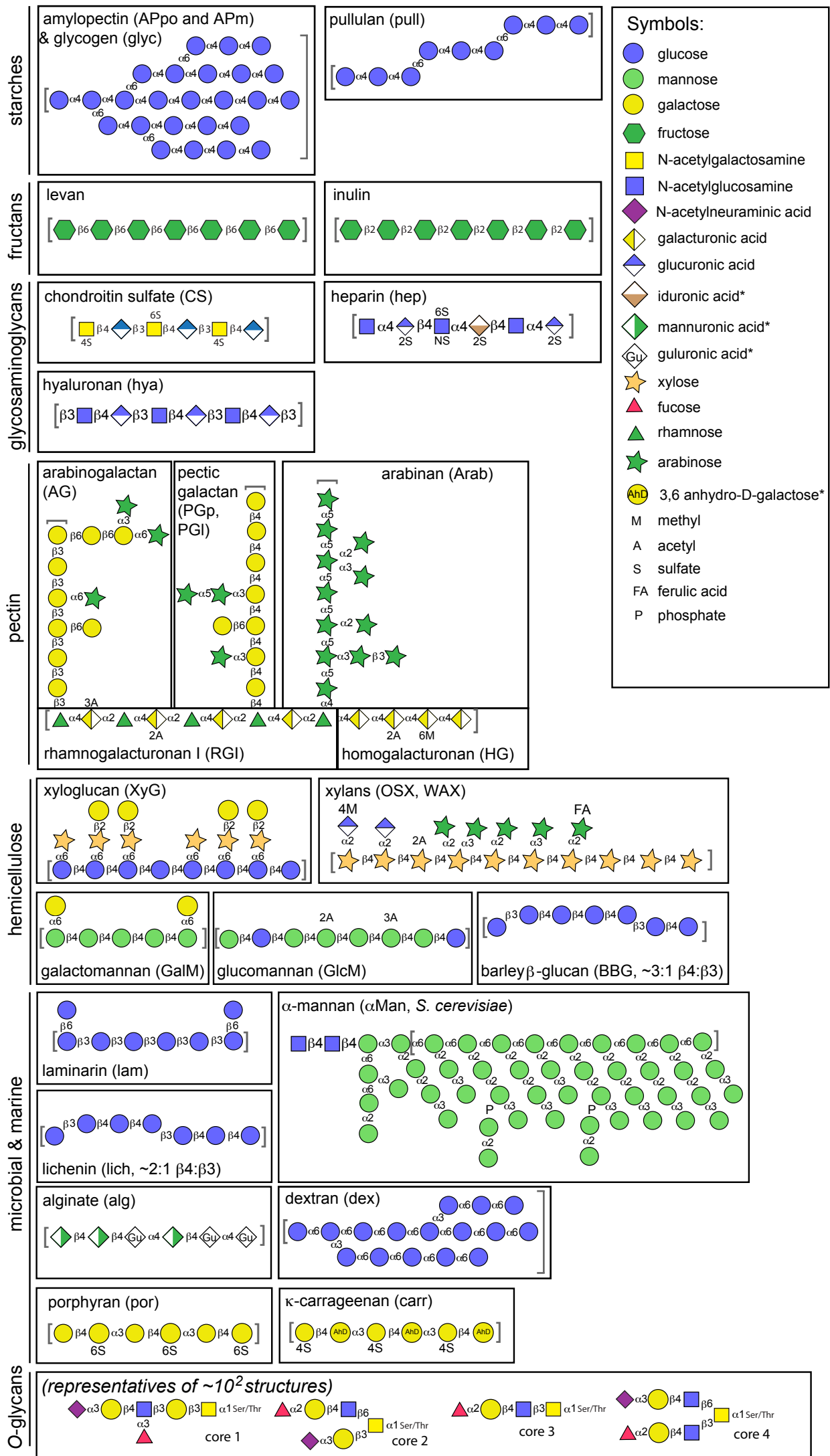

Supplement: FIG S1 [file msystems.00947-21-sf001.pdf]

Figure S3

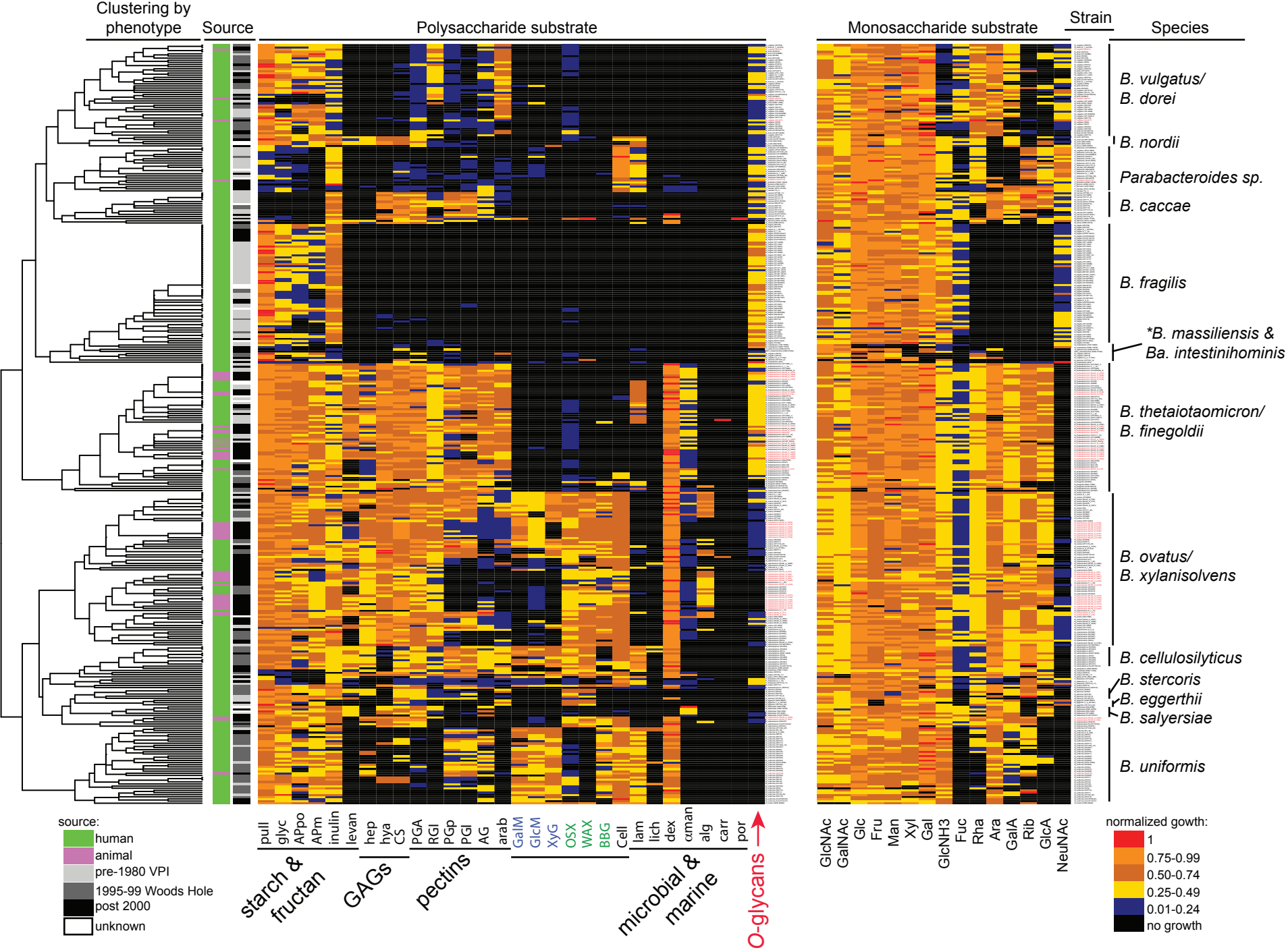

Supplement: FIG S3 [file msystems.00947-21-sf003.pdf]

Figure S5

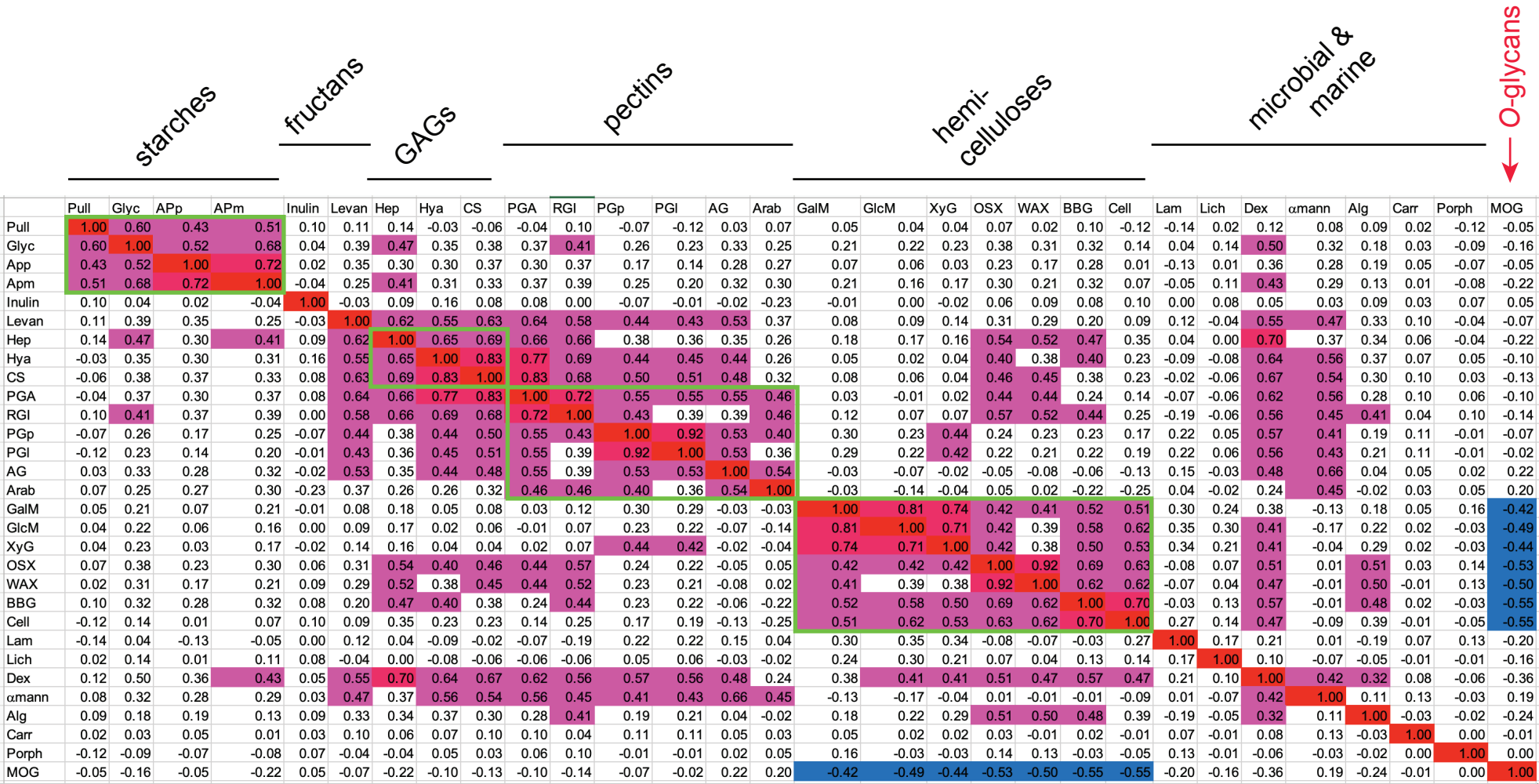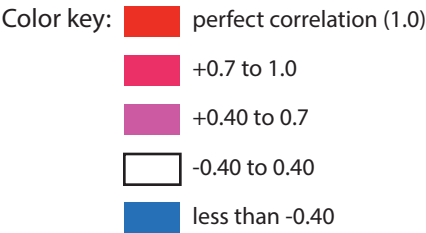

Supplement: FIG S5 [file msystems.00947-21-sf005.pdf]

Figure S6

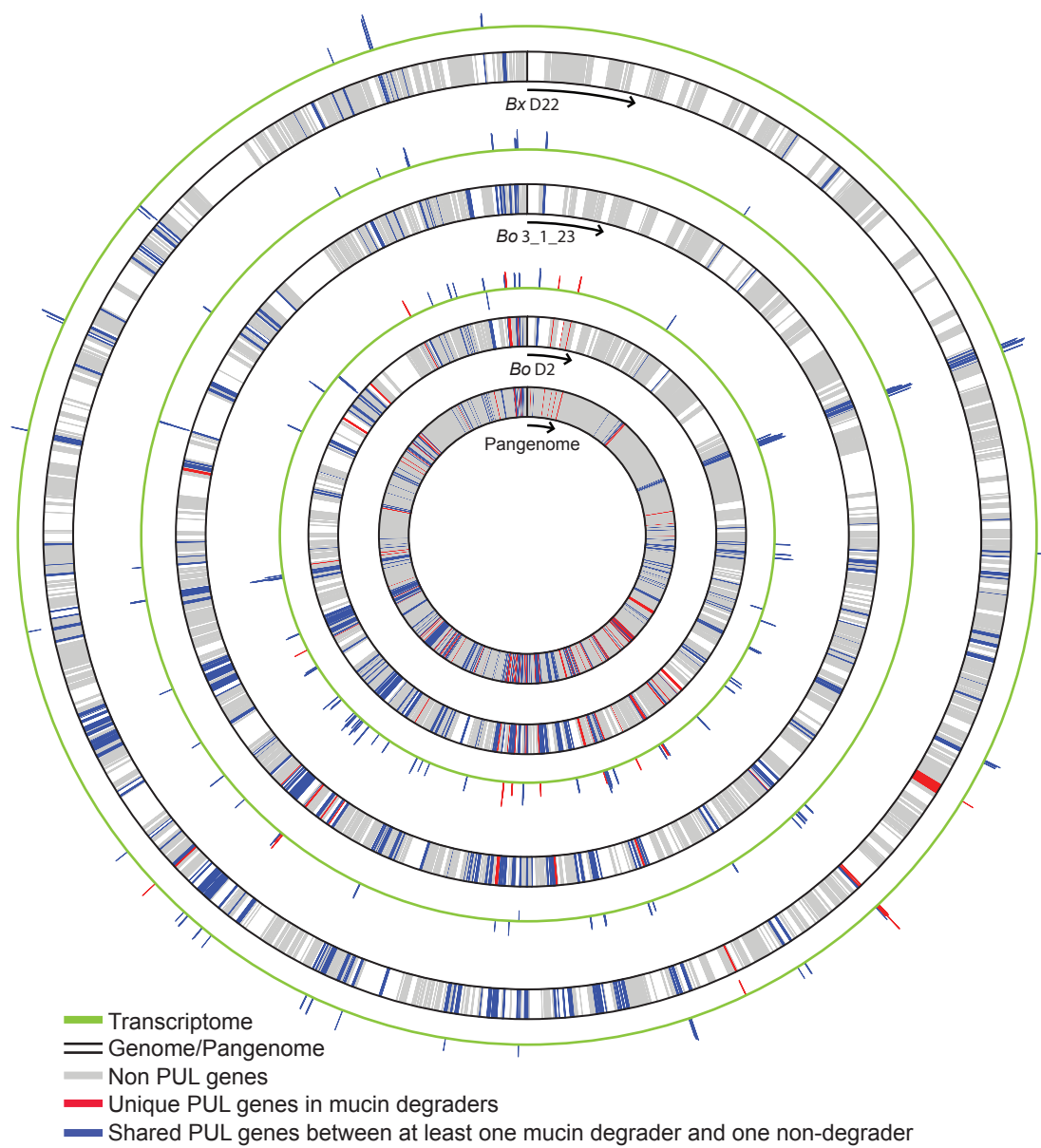

Supplement: FIG S6 [file msystems.00947-21-sf006.pdf]
