# Supplementary material for: Phenotypic and Genomic Diversification in Complex Carbohydrate-Degrading Human Gut Bacteria
Source: mSystems. 2022 Feb 15;7(1):e00947-21. doi: 10.1128/msystems.00947-21 (PMC8845570; doi:10.1128/msystems.00947-21)
Supplement: FIG S2 [file msystems.00947-21-sf002.pdf]

Figure S2

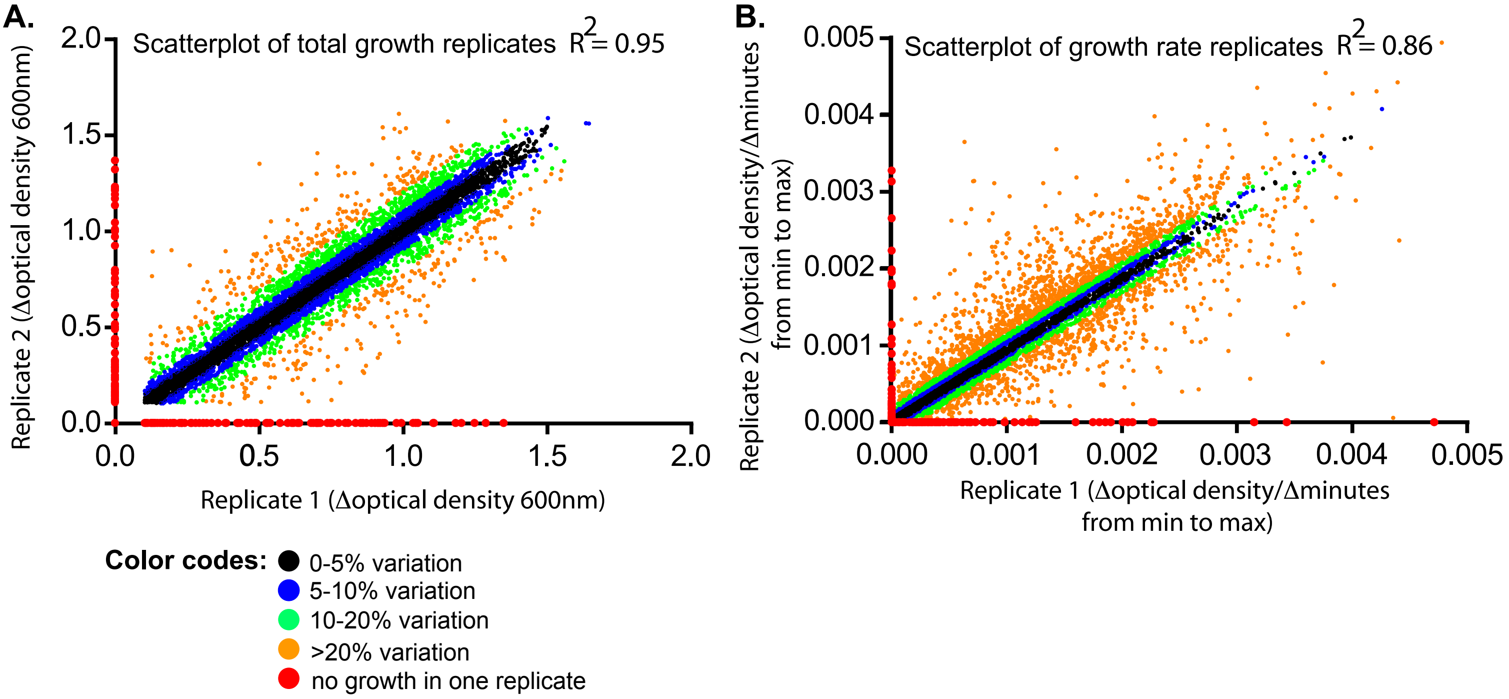

C.

Polysaccharides:

Substrate	R^2 between growth values	R^2 between rate values
AG	0.99	0.89
alg	0.96	0.96
α-mann	0.93	0.76
APm	0.94	0.92
APpo	0.96	0.91
arab	0.98	0.96
BBG	0.95	0.70
carr	0.93	0.96
Cell	0.96	0.81
CS	0.96	0.91
dex	0.96	0.87
GalM	0.96	0.98
GlcM	0.93	0.85
glyc	0.96	0.94
hep	0.96	0.85
hya	0.91	0.88
inulin	0.92	0.89
lam	0.96	0.96
levan	0.96	0.88
lich	0.80	0.45
MOG	0.98	0.97
OSX	0.93	0.83
PGA	0.97	0.89
PGI	0.95	0.96
PGp	0.92	0.92
por	0.85	0.85
pull	0.84	0.78
RGI	0.96	0.98
WAX	0.97	0.42
XyG	0.92	0.72

Monosaccharides:

Substrate	R^2 between growth values
Ara	0.90
Fru	0.90
Fuc	0.93
Gal	0.60
GalA	0.86
GalNAc	0.93
Glc	0.69
GlcA	0.87
GlcNAc	0.72
GlcNH3	0.93
Man	0.88
NeuNAc	0.86
Rha	0.96
Rib	0.94
Xyl	0.85
