# Supplementary material for: Phenotypic and Genomic Diversification in Complex Carbohydrate-Degrading Human Gut Bacteria
Source: mSystems. 2022 Feb 15;7(1):e00947-21. doi: 10.1128/msystems.00947-21 (PMC8845570; doi:10.1128/msystems.00947-21)
Supplement: FIG S4 [file msystems.00947-21-sf004.pdf]

Figure S4

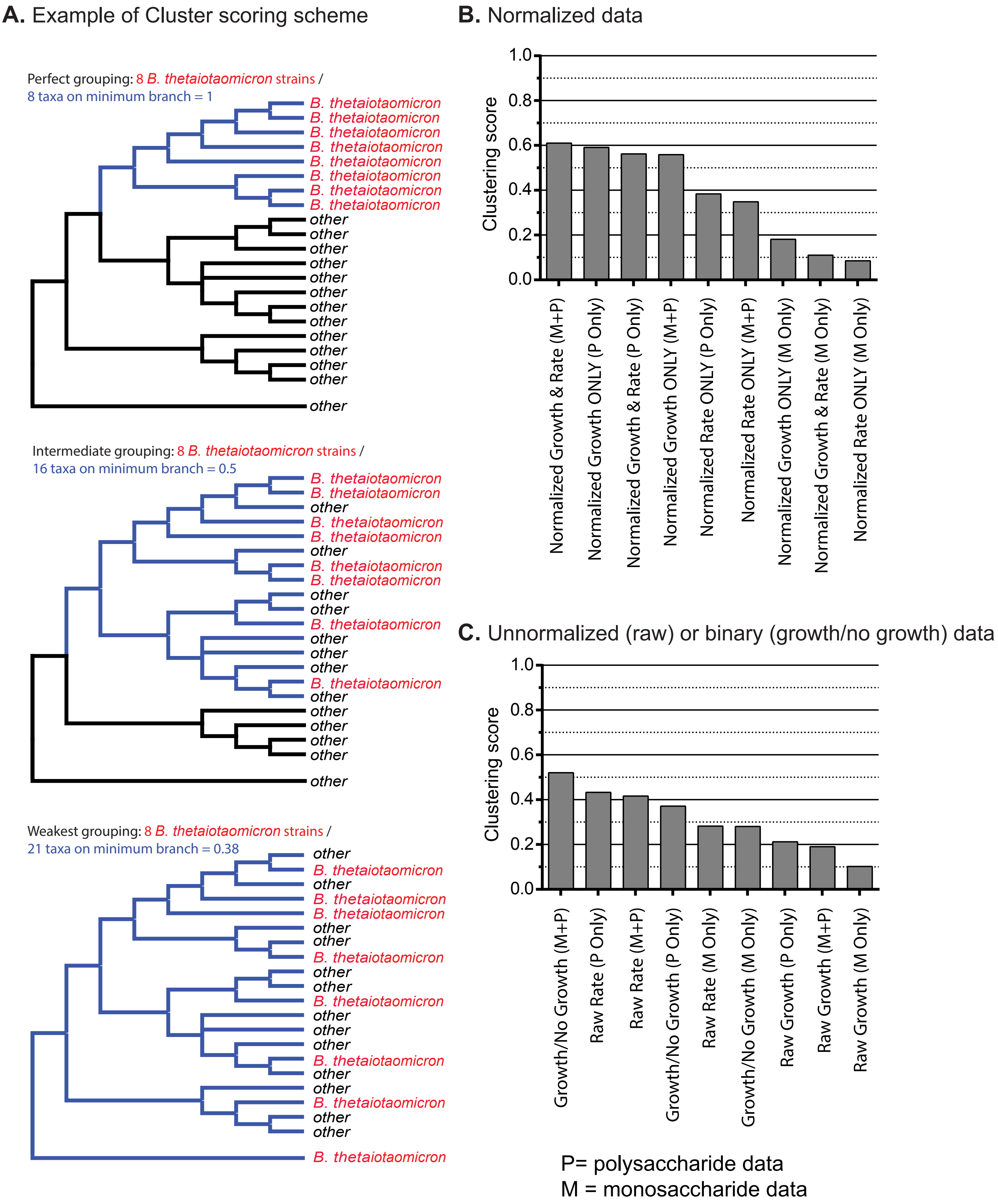
A. Example of Cluster scoring scheme
Perfect grouping: 8 B. thetaiotaomicron strains /
8 taxa on minimum branch = 1
B. thetaiotaomicron
other
Intermediate grouping: 8 B. thetaiotaomicron strains /
16 taxa on minimum branch = 0.5
Weakest grouping: 8 B. thetaiotaomicron strains /
21 taxa on minimum branch = 0.38
B. Normalized data
Clustering score
1.0
0.8
0.6
0.4
0.2
0.0
Normalized Growth & Rate (M+P)
Normalized Growth ONLY (P Only)
Normalized Growth & Rate (P Only)
Normalized Growth ONLY (M+P)
Normalized Rate ONLY (P Only)
Normalized Rate ONLY (M+P)
Normalized Growth ONLY (M Only)
Normalized Growth & Rate (M Only)
Normalized Rate ONLY (M Only)
C. Unnormalized (raw) or binary (growth/no growth) data
Clustering score
1.0
0.8
0.6
0.4
0.2
0.0
Growth/No Growth (M+P)
Raw Rate (P Only)
Raw Rate (M+P)
Growth/No Growth (P Only)
Raw Rate (M Only)
Growth/No Growth (M Only)
Raw Growth (P Only)
Raw Growth (M+P)
Raw Growth (M Only)
P= polysaccharide data
M = monosaccharide data
