# Supplementary material for: Phenotypic and Genomic Diversification in Complex Carbohydrate-Degrading Human Gut Bacteria
Source: mSystems. 2022 Feb 15;7(1):e00947-21. doi: 10.1128/msystems.00947-21 (PMC8845570; doi:10.1128/msystems.00947-21)
Supplement: FIG S7 [file msystems.00947-21-sf007.pdf]

Supplemental Figure 7. Bacteroides LGT Loci

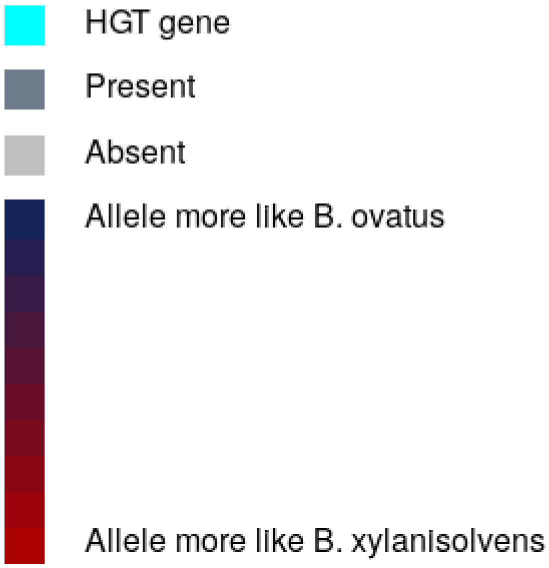

PUL LGT Events

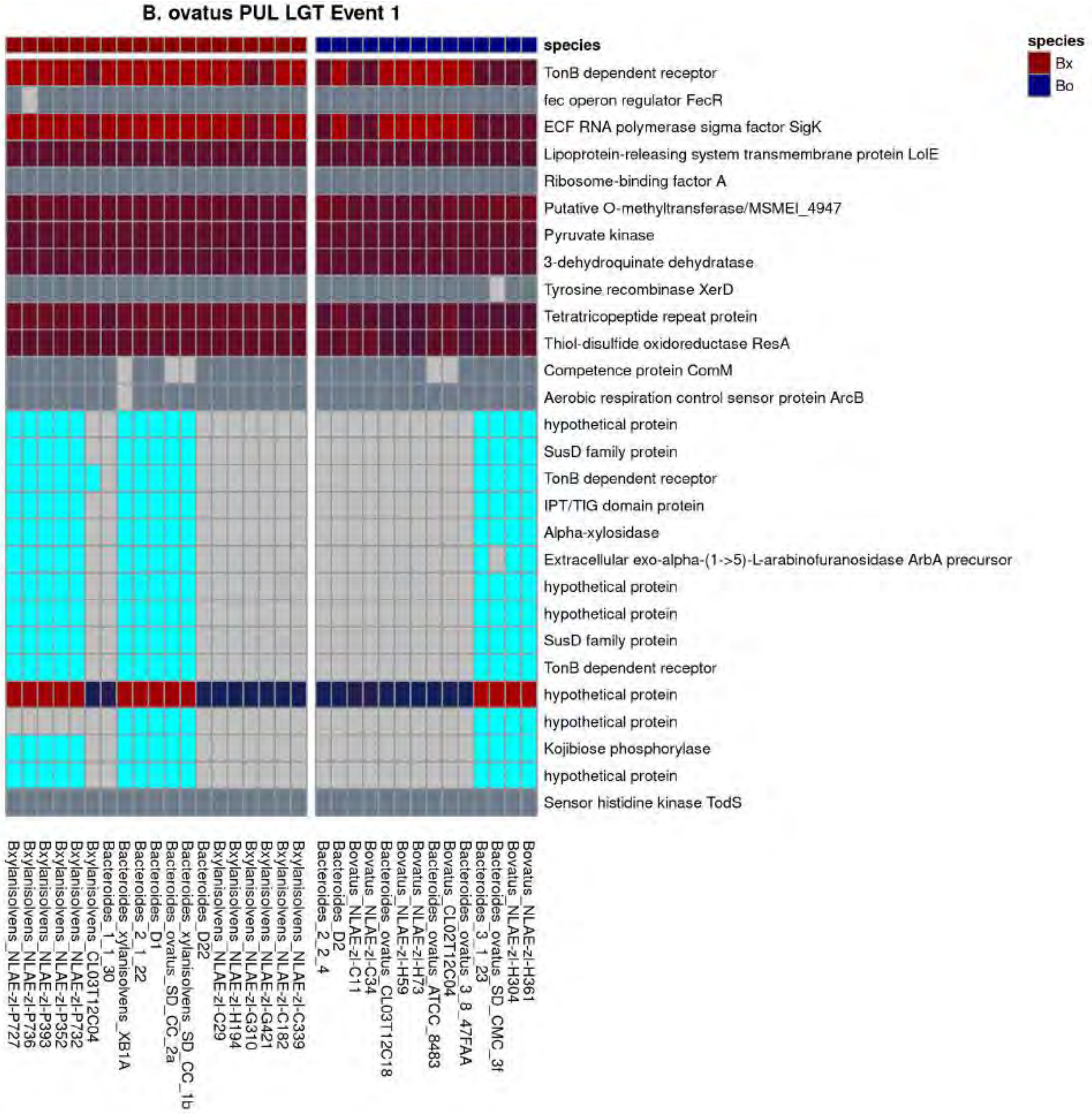

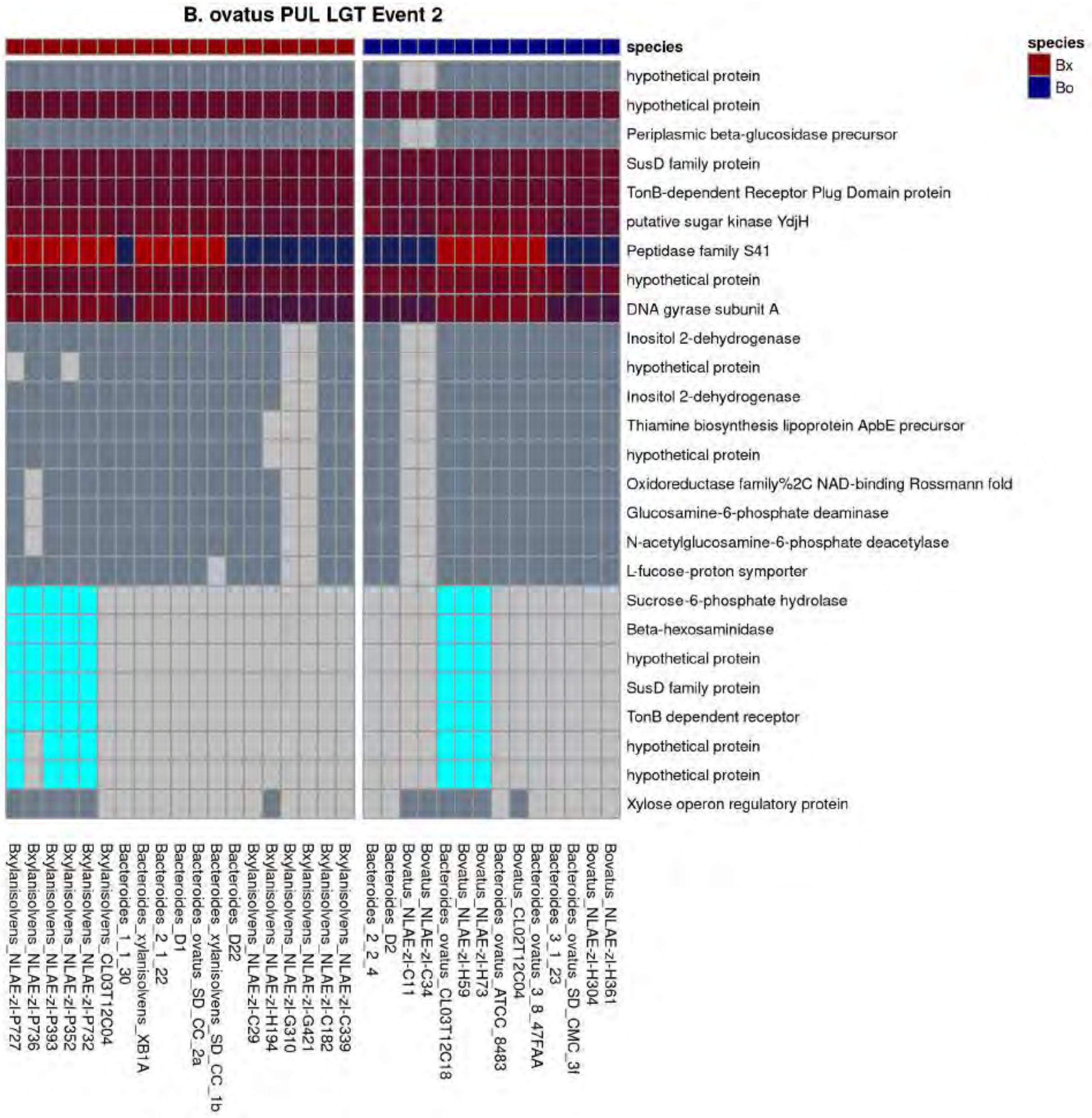
B. ovatus PUL LGT Event 2
species
Bx
Bo
hypothetical protein
hypothetical protein
Periplasmic beta-glucosidase precursor
SusD family protein
TonB-dependent Receptor Plug Domain protein
putative sugar kinase YdjH
Peptidase family S41
hypothetical protein
DNA gyrase subunit A
Inositol 2-dehydrogenase
hypothetical protein
Inositol 2-dehydrogenase
Thiamine biosynthesis lipoprotein ApbE precursor
hypothetical protein
Oxidoreductase family%2C NAD-binding Rossmann fold
Glucosamine-6-phosphate deaminase
N-acetylglucosamine-6-phosphate deacetylase
L-fucose-proton symporter
Sucrose-6-phosphate hydrolase
Beta-hexosaminidase
hypothetical protein
SusD family protein
TonB dependent receptor
hypothetical protein
hypothetical protein
Xylose operon regulatory protein
Bxylanisolvens_NLAE-zl-P727
Bxylanisolvens_NLAE-zl-P736
Bxylanisolvens_NLAE-zl-P393
Bxylanisolvens_NLAE-zl-P352
Bxylanisolvens_NLAE-zl-P732
Bxylanisolvens_CL03T12C04
Bacteroides_1_1_30
Bacteroides_xylanisolvens_XB1A
Bacteroides_2_1_22
Bacteroides_D1
Bacteroides_ovatus_SD_CC_2a
Bacteroides_xylanisolvens_SD_CC_1b
Bacteroides_D22
Bxylanisolvens_NLAE-zl-C29
Bxylanisolvens_NLAE-zl-H194
Bxylanisolvens_NLAE-zl-G310
Bxylanisolvens_NLAE-zl-G421
Bxylanisolvens_NLAE-zl-C182
Bxylanisolvens_NLAE-zl-C339
Bacteroides_2_2_4
Bacteroides_D2
Bovatus_NLAE-zl-C11
Bovatus_NLAE-zl-C34
Bacteroides_ovatus_CL03T12C18
Bovatus_NLAE-zl-H59
Bovatus_NLAE-zl-H73
Bacteroides_ovatus_ATCC_8483
Bovatus_CL02T12C04
Bacteroides_ovatus_3_8_47FAA
Bacteroides_3_1_23
Bacteroides_ovatus_SD_CMC_3f
Bovatus_NLAE-zl-H304
Bovatus_NLAE-zl-H361

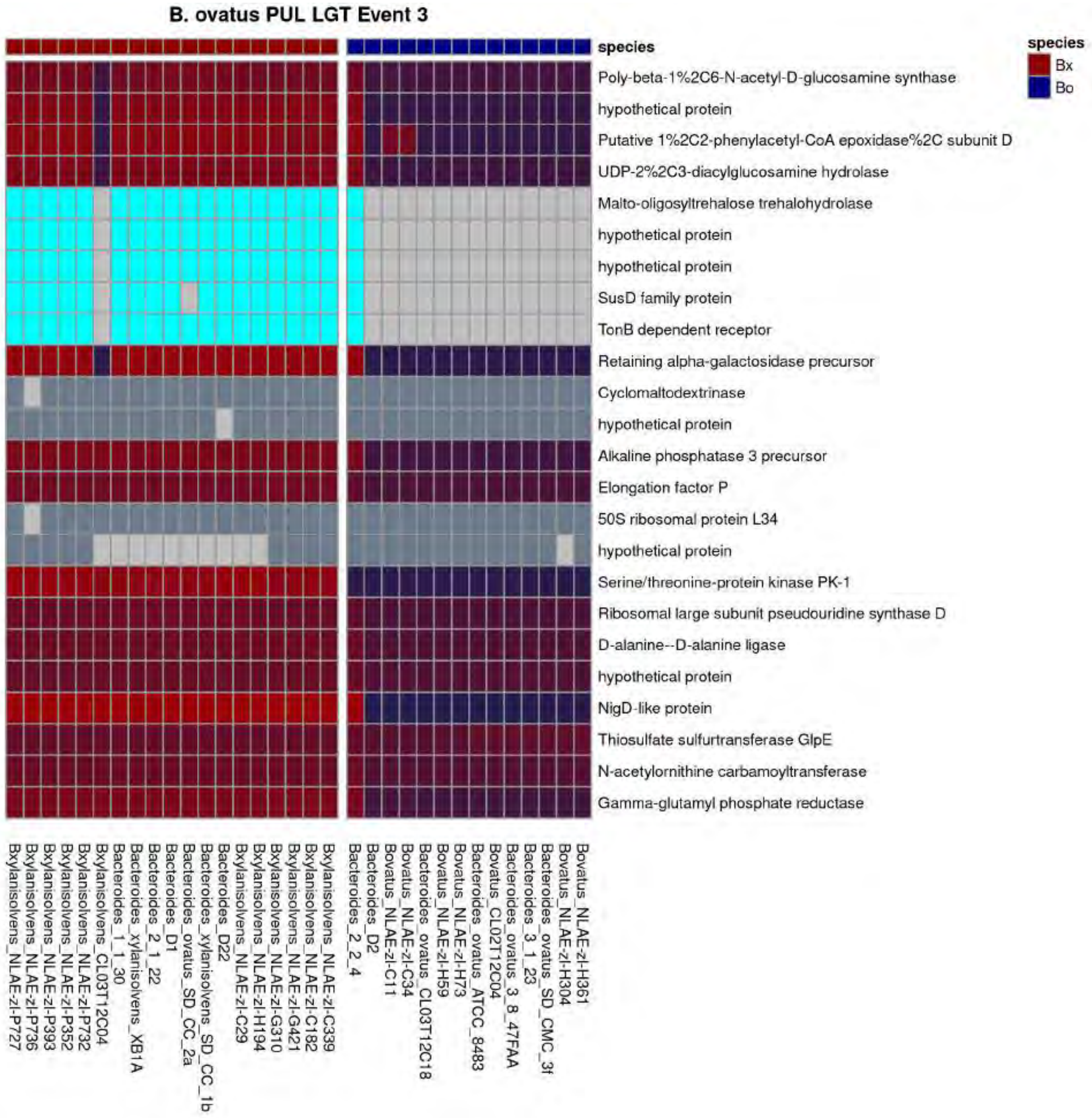

B. ovatus PUL LGT Event 3
species
Poly-beta-1%2C6-N-acetyl-D-glucosamine synthase
hypothetical protein
Putative 1%2C2-phenylacetyl-CoA epoxidase%2C subunit D
UDP-2%2C3-diacylglucosamine hydrolase
Malto-oligosyltrehalose trehalohydrolase
hypothetical protein
hypothetical protein
SusD family protein
TonB dependent receptor
Retaining alpha-galactosidase precursor
Cyclomaltodextrinase
hypothetical protein
Alkaline phosphatase 3 precursor
Elongation factor P
50S ribosomal protein L34
hypothetical protein
Serine/threonine-protein kinase PK-1
Ribosomal large subunit pseudouridine synthase D
D-alanine--D-alanine ligase
hypothetical protein
NigD-like protein
Thiosulfate sulfurtransferase GlpE
N-acetylornithine carbamoyltransferase
Gamma-glutamyl phosphate reductase
species
Bx
Bo
Bxylanisolvens_NLAE-zl-P727
Bxylanisolvens_NLAE-zl-P736
Bxylanisolvens_NLAE-zl-P393
Bxylanisolvens_NLAE-zl-P352
Bxylanisolvens_NLAE-zl-P732
Bxylanisolvens_CL03T12C04
Bacteroides_1_1_30
Bacteroides_xylanisolvens_XB1A
Bacteroides_2_1_22
Bacteroides_D1
Bacteroides_ovatus_SD_CC_2a
Bacteroides_xylanisolvens_SD_CC_1b
Bacteroides_D22
Bxylanisolvens_NLAE-zl-C29
Bxylanisolvens_NLAE-zl-H194
Bxylanisolvens_NLAE-zl-G310
Bxylanisolvens_NLAE-zl-G421
Bxylanisolvens_NLAE-zl-C182
Bxylanisolvens_NLAE-zl-C339
Bacteroides_2_2_4
Bacteroides_D2
Bovatus_NLAE-zl-C11
Bovatus_NLAE-zl-C34
Bacteroides_ovatus_CL03T12C18
Bovatus_NLAE-zl-H59
Bovatus_NLAE-zl-H73
Bacteroides_ovatus_ATCC_8483
Bovatus_CL02T12C04
Bacteroides_ovatus_3_8_47FAA
Bacteroides_3_1_23
Bacteroides_ovatus_SD_CMC_3f
Bovatus_NLAE-zl-H304
Bovatus_NLAE-zl-H361

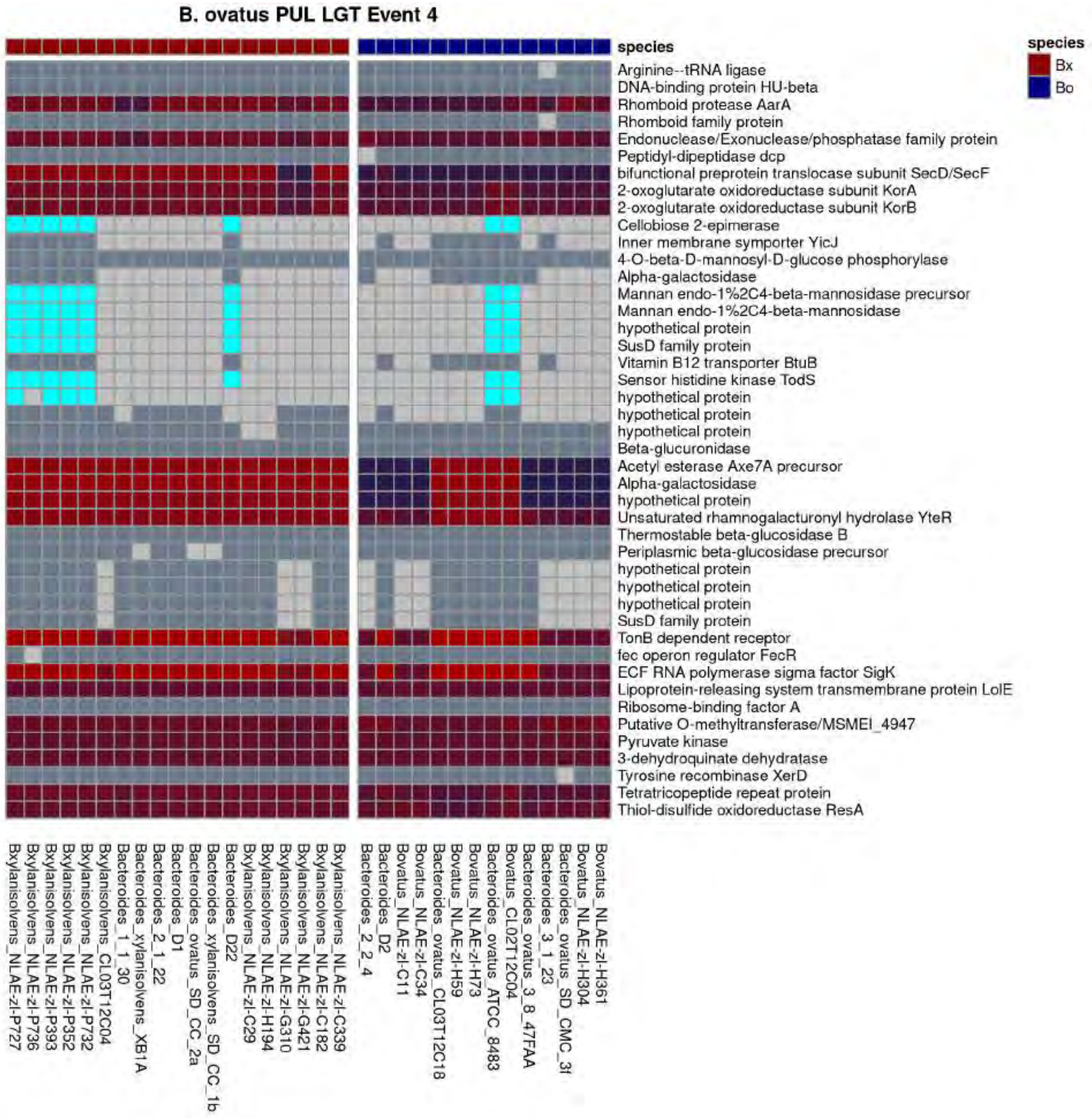

B. ovatus PUL LGT Event 4
species
Bx
Bo
Arginine--tRNA ligase
DNA-binding protein HU-beta
Rhomboid protease AarA
Rhomboid family protein
Endonuclease/Exonuclease/phosphatase family protein
Peptidyl-dipeptidase dcp
bifunctional preprotein translocase subunit SecD/SecF
2-oxoglutarate oxidoreductase subunit KorA
2-oxoglutarate oxidoreductase subunit KorB
Cellobiose 2-epimerase
Inner membrane symporter YicJ
4-O-beta-D-mannosyl-D-glucose phosphorylase
Alpha-galactosidase
Mannan endo-1%2C4-beta-mannosidase precursor
Mannan endo-1%2C4-beta-mannosidase
hypothetical protein
SusD family protein
Vitamin B12 transporter BtuB
Sensor histidine kinase TodS
hypothetical protein
hypothetical protein
hypothetical protein
Beta-glucuronidase
Acetyl esterase Axe7A precursor
Alpha-galactosidase
hypothetical protein
Unsaturated rhamnogalacturonyl hydrolase YteR
Thermostable beta-glucosidase B
Periplasmic beta-glucosidase precursor
hypothetical protein
hypothetical protein
hypothetical protein
SusD family protein
TonB dependent receptor
fec operon regulator FecR
ECF RNA polymerase sigma factor SigK
Lipoprotein-releasing system transmembrane protein LolE
Ribosome-binding factor A
Putative O-methyltransferase/MSMEI_4947
Pyruvate kinase
3-dehydroquinate dehydratase
Tyrosine recombinase XerD
Tetratricopeptide repeat protein
Thiol-disulfide oxidoreductase ResA
Bxylanisolvens_NLAE-zl-P727
Bxylanisolvens_NLAE-zl-P736
Bxylanisolvens_NLAE-zl-P393
Bxylanisolvens_NLAE-zl-P352
Bxylanisolvens_NLAE-zl-P732
Bxylanisolvens_CL03T12C04
Bacteroides_1_1_30
Bacteroides_xylanisolvens_XB1A
Bacteroides_2_1_22
Bacteroides_D1
Bacteroides_ovatus_SD_CC_2a
Bacteroides_xylanisolvens_SD_CC_1b
Bacteroides_D22
Bxylanisolvens_NLAE-zl-C29
Bxylanisolvens_NLAE-zl-H194
Bxylanisolvens_NLAE-zl-G310
Bxylanisolvens_NLAE-zl-G421
Bxylanisolvens_NLAE-zl-C182
Bxylanisolvens_NLAE-zl-C339
Bacteroides_2_2_4
Bacteroides_D2
Bovatus_NLAE-zl-C11
Bovatus_NLAE-zl-C34
Bacteroides_ovatus_CL03T12C18
Bovatus_NLAE-zl-H59
Bovatus_NLAE-zl-H73
Bacteroides_ovatus_ATCC_8483
Bovatus_CL02T12C04
Bacteroides_ovatus_3_8_47FAA
Bacteroides_3_1_23
Bacteroides_ovatus_SD_CMC_3f
Bovatus_NLAE-zl-H304
Bovatus_NLAE-zl-H361

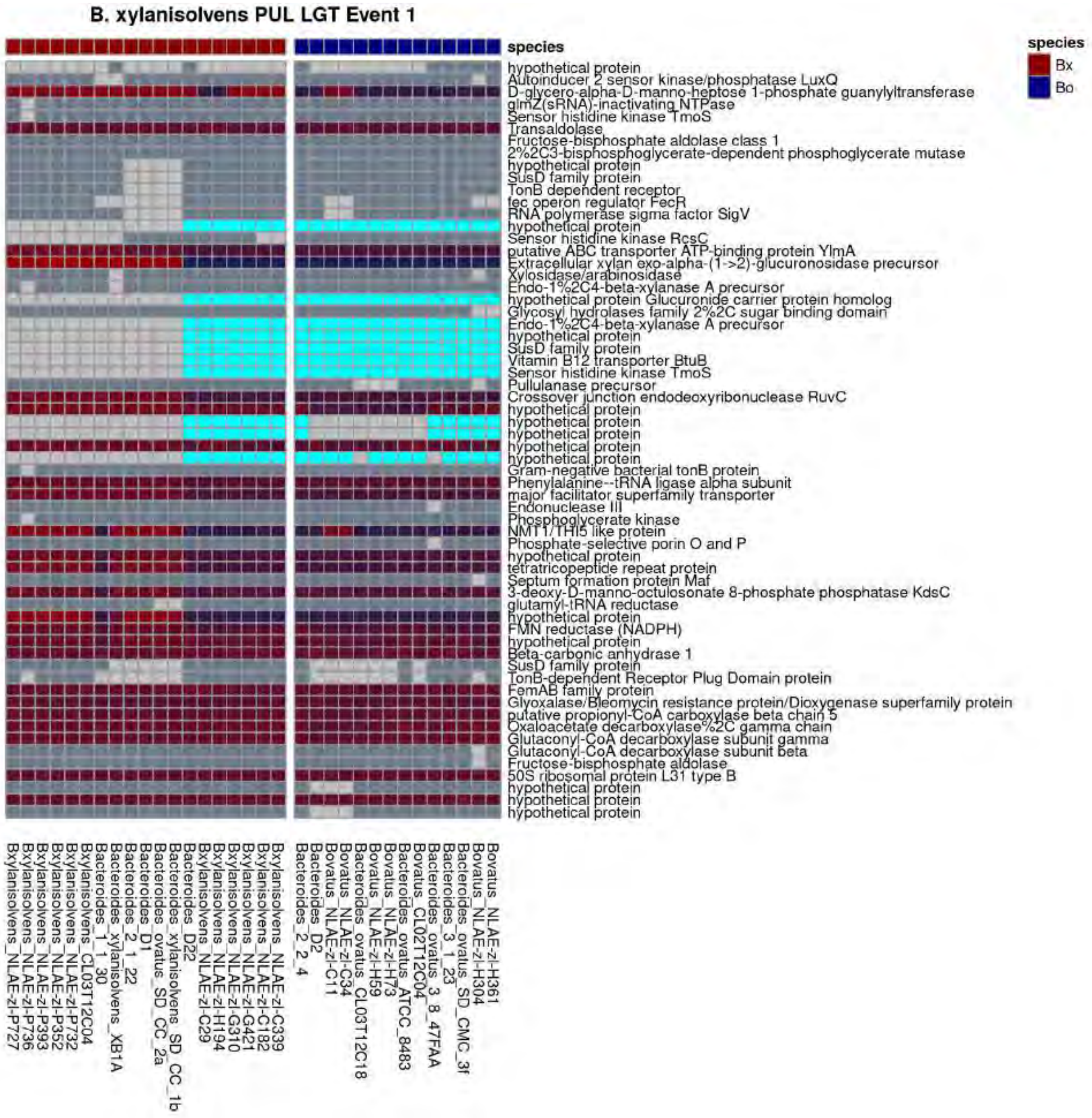
B. xylanisolvens PUL LGT Event 1
species
species
Bx
Bo
hypothetical protein
Autoinducer 2 sensor kinase/phosphatase LuxQ
D-glycero-alpha-D-manno-heptose 1-phosphate guanylyltransferase
glmZ(sRNA)-inactivating NTPase
Sensor histidine kinase TmoS
Transaldolase
Fructose-bisphosphate aldolase class 1
2%2C3-bisphosphoglycerate-dependent phosphoglycerate mutase
hypothetical protein
SusD family protein
TonB dependent receptor
fec operon regulator FecR
RNA polymerase sigma factor SigV
hypothetical protein
Sensor histidine kinase RcsC
putative ABC transporter ATP-binding protein YlmA
Extracellular xylan exo-alpha-(1->2)-glucuronosidase precursor
Xylosidase/arabinosidase
Endo-1%2C4-beta-xylanase A precursor
hypothetical protein Glucuronide carrier protein homolog
Glycosyl hydrolases family 2%2C sugar binding domain
Endo-1%2C4-beta-xylanase A precursor
hypothetical protein
SusD family protein
Vitamin B12 transporter BtuB
Sensor histidine kinase TmoS
Pullulanase precursor
Crossover junction endodeoxyribonuclease RuvC
hypothetical protein
hypothetical protein
hypothetical protein
hypothetical protein
hypothetical protein
Gram-negative bacterial tonB protein
Phenylalanine--tRNA ligase alpha subunit
major facilitator superfamily transporter
Endonuclease III
Phosphoglycerate kinase
NMT1/THI5 like protein
Phosphate-selective porin O and P
hypothetical protein
tetratricopeptide repeat protein
Septum formation protein Maf
3-deoxy-D-manno-octulosonate 8-phosphate phosphatase KdsC
glutamyl-tRNA reductase
hypothetical protein
FMN reductase (NADPH)
hypothetical protein
Beta-carbonic anhydrase 1
SusD family protein
TonB-dependent Receptor Plug Domain protein
FemAB family protein
Glyoxalase/Bleomycin resistance protein/Dioxygenase superfamily protein
putative propionyl-CoA carboxylase beta chain 5
Oxaloacetate decarboxylase%2C gamma chain
Glutaconyl-CoA decarboxylase subunit gamma
Glutaconyl-CoA decarboxylase subunit beta
Fructose-bisphosphate aldolase
50S ribosomal protein L31 type B
hypothetical protein
hypothetical protein
hypothetical protein
Bxylanisolvens_NLAE-zl-P727
Bxylanisolvens_NLAE-zl-P736
Bxylanisolvens_NLAE-zl-P393
Bxylanisolvens_NLAE-zl-P352
Bxylanisolvens_NLAE-zl-P732
Bxylanisolvens_CL03T12C04
Bacteroides_1_1_30
Bacteroides_xylanisolvens_XB1A
Bacteroides_2_1_22
Bacteroides_D1
Bacteroides_ovatus_SD_CC_2a
Bacteroides_xylanisolvens_SD_CC_1b
Bacteroides_D22
Bxylanisolvens_NLAE-zl-C29
Bxylanisolvens_NLAE-zl-H194
Bxylanisolvens_NLAE-zl-G310
Bxylanisolvens_NLAE-zl-G421
Bxylanisolvens_NLAE-zl-C182
Bxylanisolvens_NLAE-zl-C339
Bacteroides_2_2_4
Bacteroides_D2
Bovatus_NLAE-zl-C11
Bovatus_NLAE-zl-C34
Bacteroides_ovatus_CL03T12C18
Bovatus_NLAE-zl-H59
Bovatus_NLAE-zl-H73
Bacteroides_ovatus_ATCC_8483
Bovatus_CL02T12C04
Bacteroides_ovatus_3_8_47FAA
Bacteroides_3_1_23
Bacteroides_ovatus_SD_CMC_3f
Bovatus_NLAE-zl-H304
Bovatus_NLAE-zl-H361

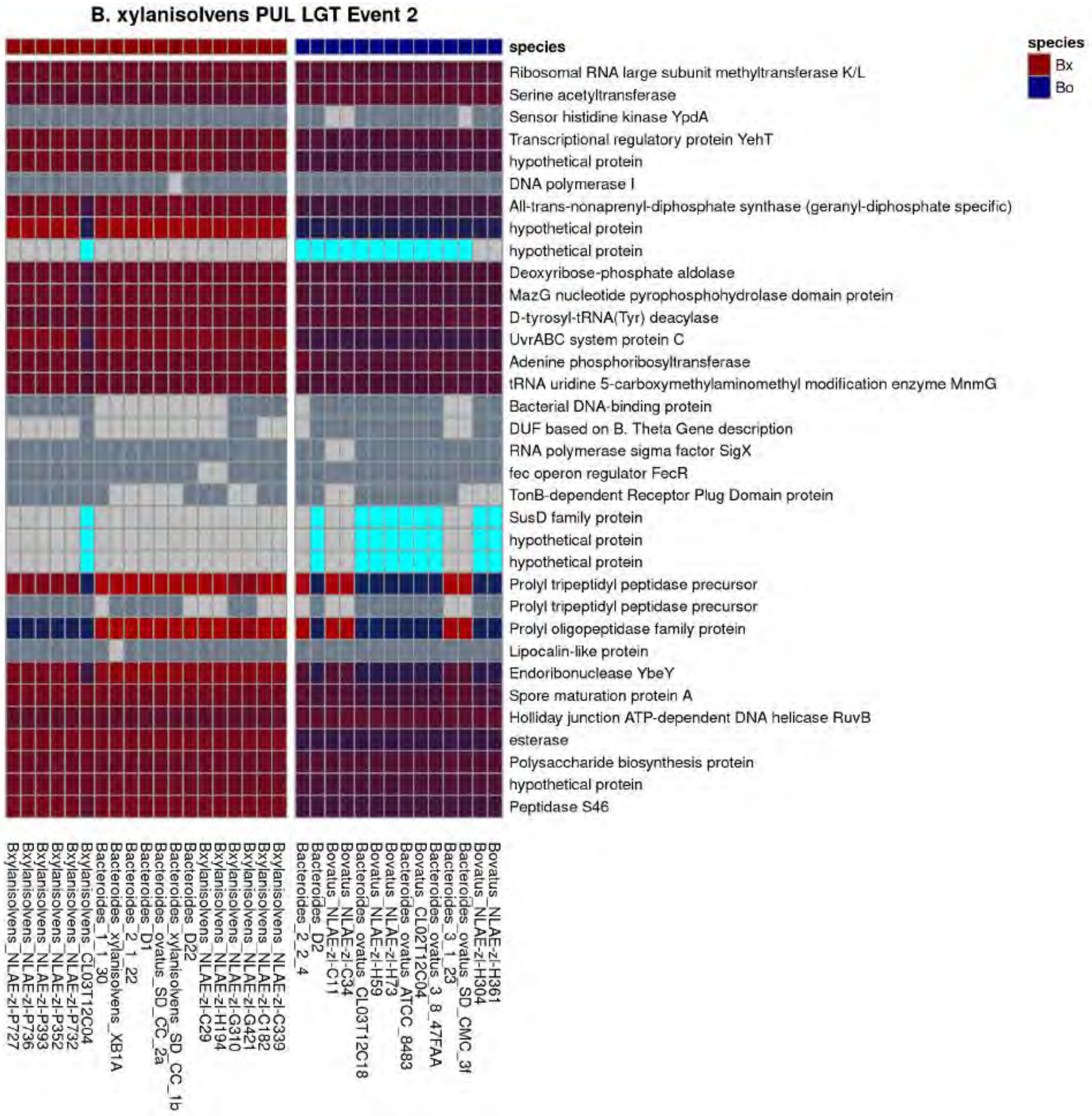

B. xylanisolvens PUL LGT Event 2
species
Ribosomal RNA large subunit methyltransferase K/L
Serine acetyltransferase
Sensor histidine kinase YpdA
Transcriptional regulatory protein YehT
hypothetical protein
DNA polymerase I
All-trans-nonaprenyl-diphosphate synthase (geranyl-diphosphate specific)
hypothetical protein
hypothetical protein
Deoxyribose-phosphate aldolase
MazG nucleotide pyrophosphohydrolase domain protein
D-tyrosyl-tRNA(Tyr) deacylase
UvrABC system protein C
Adenine phosphoribosyltransferase
tRNA uridine 5-carboxymethylaminomethyl modification enzyme MnmG
Bacterial DNA-binding protein
DUF based on B. Theta Gene description
RNA polymerase sigma factor SigX
fec operon regulator FecR
TonB-dependent Receptor Plug Domain protein
SusD family protein
hypothetical protein
hypothetical protein
Prolyl tripeptidyl peptidase precursor
Prolyl tripeptidyl peptidase precursor
Prolyl oligopeptidase family protein
Lipocalin-like protein
Endoribonuclease YbeY
Spore maturation protein A
Holliday junction ATP-dependent DNA helicase RuvB
esterase
Polysaccharide biosynthesis protein
hypothetical protein
Peptidase S46
species
Bx
Bo
Bxylanisolvens_NLAE-zl-P727
Bxylanisolvens_NLAE-zl-P736
Bxylanisolvens_NLAE-zl-P393
Bxylanisolvens_NLAE-zl-P352
Bxylanisolvens_NLAE-zl-P732
Bxylanisolvens_CL03T12C04
Bacteroides_1_1_30
Bacteroides_xylanisolvens_XB1A
Bacteroides_2_1_22
Bacteroides_D1
Bacteroides_ovatus_SD_CC_2a
Bacteroides_xylanisolvens_SD_CC_1b
Bacteroides_D22
Bxylanisolvens_NLAE-zl-C29
Bxylanisolvens_NLAE-zl-H194
Bxylanisolvens_NLAE-zl-G310
Bxylanisolvens_NLAE-zl-G421
Bxylanisolvens_NLAE-zl-C182
Bxylanisolvens_NLAE-zl-C339
Bacteroides_2_2_4
Bacteroides_D2
Bovatus_NLAE-zl-C11
Bovatus_NLAE-zl-C34
Bacteroides_ovatus_CL03T12C18
Bovatus_NLAE-zl-H59
Bovatus_NLAE-zl-H73
Bacteroides_ovatus_ATCC_8483
Bovatus_CL02T12C04
Bacteroides_ovatus_3_8_47FAA
Bacteroides_3_1_23
Bacteroides_ovatus_SD_CMC_3f
Bovatus_NLAE-zl-H304
Bovatus_NLAE-zl-H361

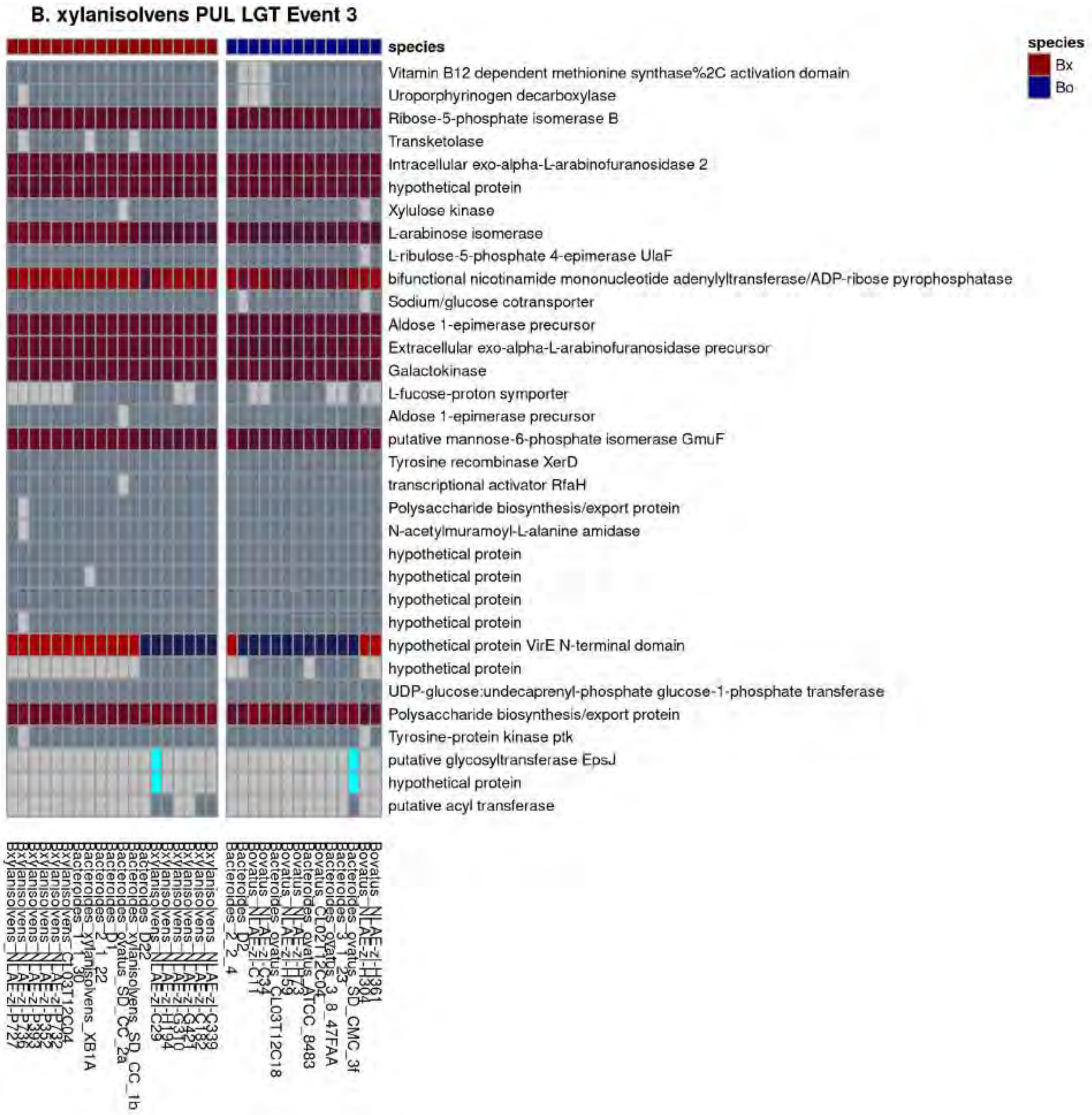
B. xylanisolvens PUL LGT Event 3
species
Vitamin B12 dependent methionine synthase%2C activation domain
Uroporphyrinogen decarboxylase
Ribose-5-phosphate isomerase B
Transketolase
Intracellular exo-alpha-L-arabinofuranosidase 2
hypothetical protein
Xylulose kinase
L-arabinose isomerase
L-ribulose-5-phosphate 4-epimerase UlaF
bifunctional nicotinamide mononucleotide adenylyltransferase/ADP-ribose pyrophosphatase
Sodium/glucose cotransporter
Aldose 1-epimerase precursor
Extracellular exo-alpha-L-arabinofuranosidase precursor
Galactokinase
L-fucose-proton symporter
Aldose 1-epimerase precursor
putative mannose-6-phosphate isomerase GmuF
Tyrosine recombinase XerD
transcriptional activator RfaH
Polysaccharide biosynthesis/export protein
N-acetylmuramoyl-L-alanine amidase
hypothetical protein
hypothetical protein
hypothetical protein
hypothetical protein
hypothetical protein VirE N-terminal domain
hypothetical protein
UDP-glucose:undecaprenyl-phosphate glucose-1-phosphate transferase
Polysaccharide biosynthesis/export protein
Tyrosine-protein kinase ptk
putative glycosyltransferase EpsJ
hypothetical protein
putative acyl transferase
species
Bx
Bo

non-PUL LGT Events

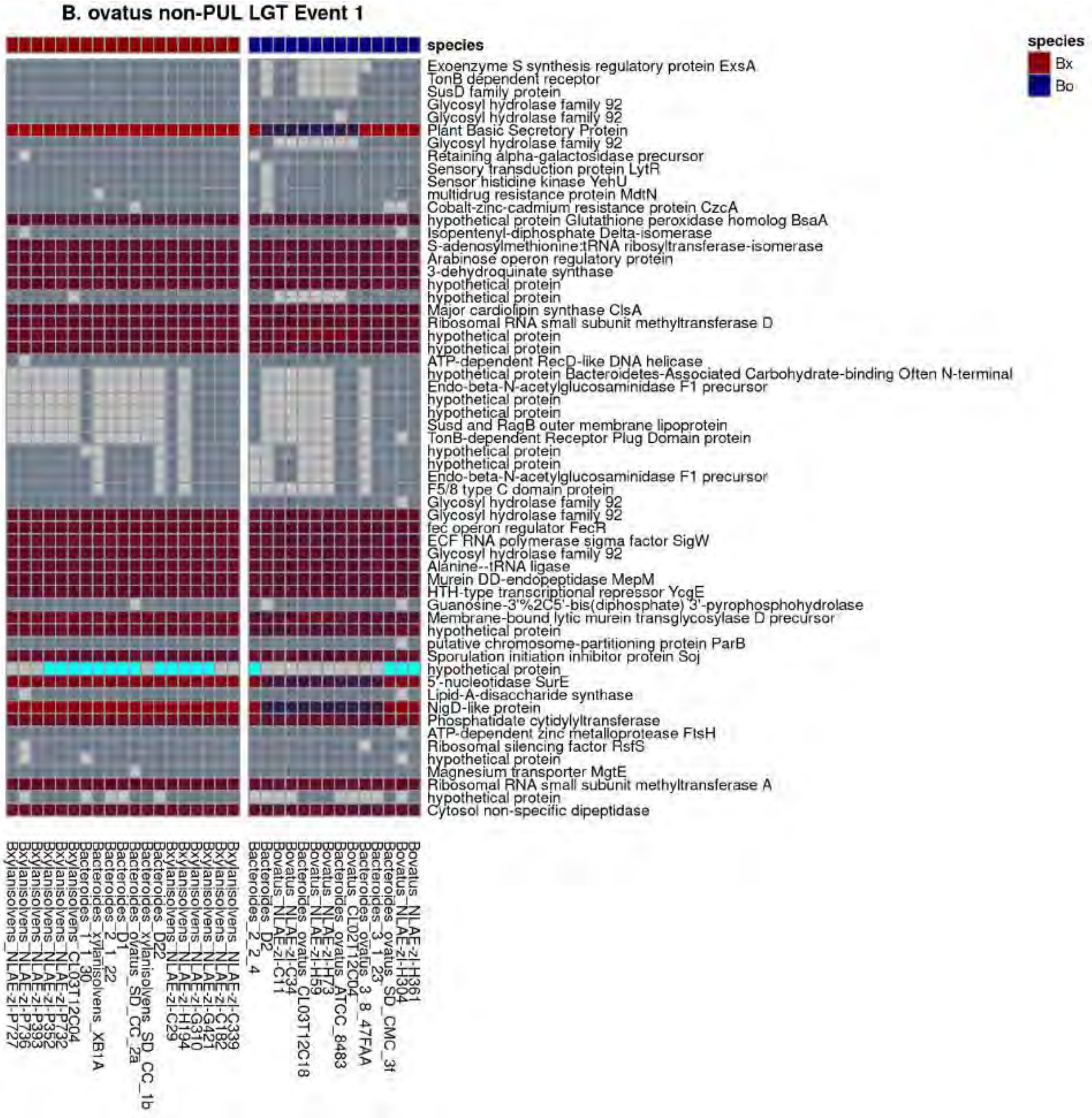

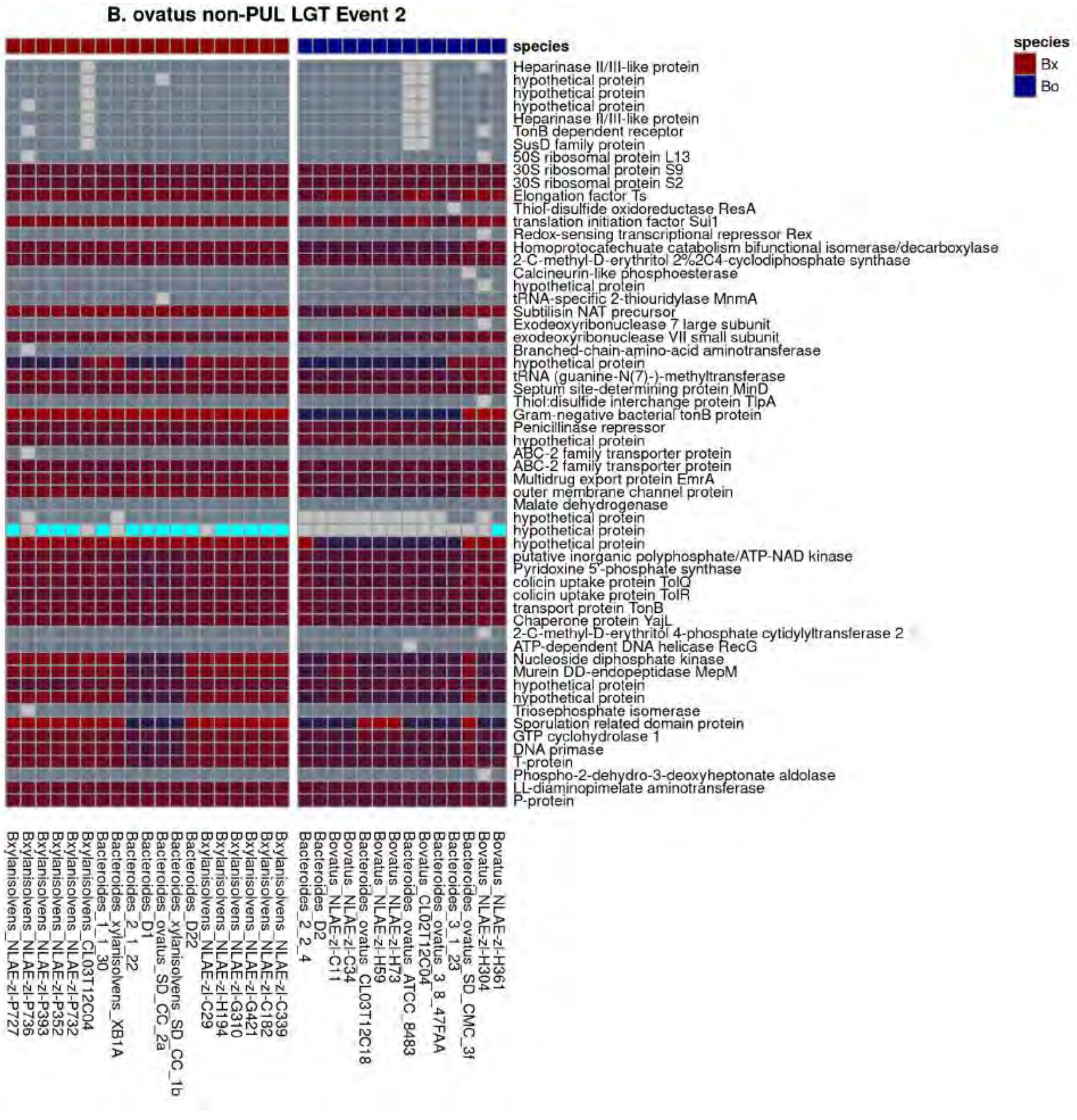
B. ovatus non-PUL LGT Event 2
species
species
Bx
Bo
Heparinase II/III-like protein
hypothetical protein
hypothetical protein
hypothetical protein
Heparinase II/III-like protein
TonB dependent receptor
SusD family protein
50S ribosomal protein L13
30S ribosomal protein S9
30S ribosomal protein S2
Elongation factor Ts
Thiol-disulfide oxidoreductase ResA
translation initiation factor Sui1
Redox-sensing transcriptional repressor Rex
Homoprotocatechuate catabolism bifunctional isomerase/decarboxylase
2-C-methyl-D-erythritol 2%2C4-cyclodiphosphate synthase
Calcineurin-like phosphoesterase
hypothetical protein
tRNA-specific 2-thiouridylase MnmA
Subtilisin NAT precursor
Exodeoxyribonuclease 7 large subunit
exodeoxyribonuclease VII small subunit
Branched-chain-amino-acid aminotransferase
hypothetical protein
tRNA (guanine-N(7)-)-methyltransferase
Septum site-determining protein MinD
Thiol:disulfide interchange protein TlpA
Gram-negative bacterial tonB protein
Penicillinase repressor
hypothetical protein
ABC-2 family transporter protein
ABC-2 family transporter protein
Multidrug export protein EmrA
outer membrane channel protein
Malate dehydrogenase
hypothetical protein
hypothetical protein
hypothetical protein
putative inorganic polyphosphate/ATP-NAD kinase
Pyridoxine 5'-phosphate synthase
colicin uptake protein TolQ
colicin uptake protein TolR
transport protein TonB
Chaperone protein YajL
2-C-methyl-D-erythritol 4-phosphate cytidylyltransferase 2
ATP-dependent DNA helicase RecG
Nucleoside diphosphate kinase
Murein DD-endopeptidase MepM
hypothetical protein
hypothetical protein
Triosephosphate isomerase
Sporulation related domain protein
GTP cyclohydrolase 1
DNA primase
T-protein
Phospho-2-dehydro-3-deoxyheptonate aldolase
LL-diaminopimelate aminotransferase
P-protein
Bxylanisolvens_NLAE-zl-P727
Bxylanisolvens_NLAE-zl-P736
Bxylanisolvens_NLAE-zl-P393
Bxylanisolvens_NLAE-zl-P352
Bxylanisolvens_NLAE-zl-P732
Bxylanisolvens_CL03T12C04
Bacteroides_1_1_30
Bacteroides_xylanisolvens_XB1A
Bacteroides_2_1_22
Bacteroides_D1
Bacteroides_xylanisolvens_SD_CC_1b
Bacteroides_ovatus_SD_CC_2a
Bacteroides_D22
Bxylanisolvens_NLAE-zl-C29
Bxylanisolvens_NLAE-zl-H194
Bxylanisolvens_NLAE-zl-G310
Bxylanisolvens_NLAE-zl-G421
Bxylanisolvens_NLAE-zl-C182
Bxylanisolvens_NLAE-zl-C339
Bacteroides_2_2_4
Bacteroides_D2
Bovatus_NLAE-zl-C11
Bovatus_NLAE-zl-C34
Bacteroides_ovatus_CL03T12C18
Bovatus_NLAE-zl-H59
Bovatus_NLAE-zl-H73
Bacteroides_ovatus_ATCC_8483
Bovatus_CL02T12C04
Bacteroides_ovatus_3_8_47FAA
Bacteroides_3_1_23
Bacteroides_ovatus_SD_CMC_3f
Bovatus_NLAE-zl-H304
Bovatus_NLAE-zl-H361

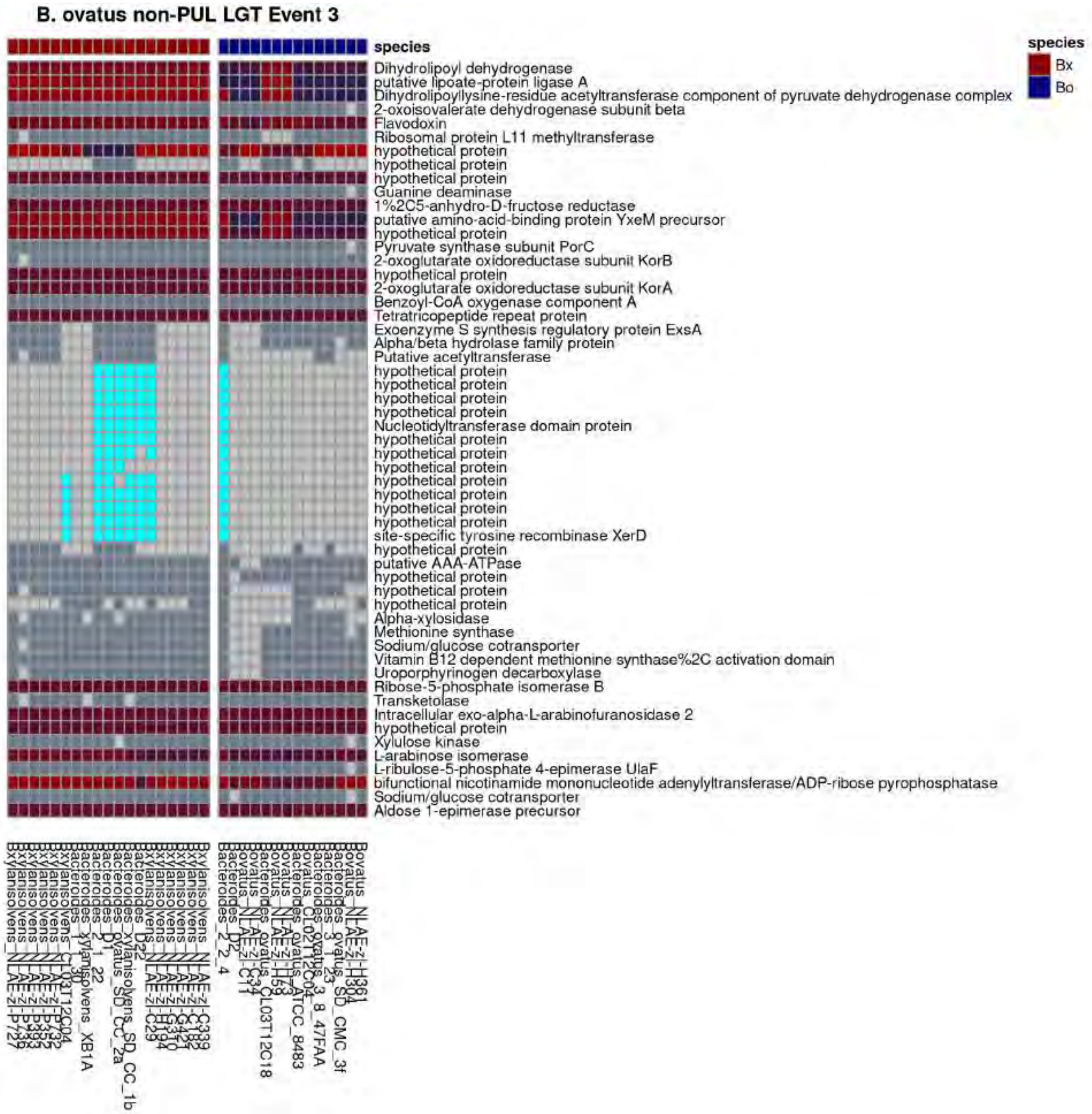
B. ovatus non-PUL LGT Event 3
species
Dihydrolipoyl dehydrogenase
putative lipoate-protein ligase A
Dihydrolipoyllysine-residue acetyltransferase component of pyruvate dehydrogenase complex
2-oxoisovalerate dehydrogenase subunit beta
Flavodoxin
Ribosomal protein L11 methyltransferase
hypothetical protein
hypothetical protein
hypothetical protein
Guanine deaminase
1%2C5-anhydro-D-fructose reductase
putative amino-acid-binding protein YxeM precursor
hypothetical protein
Pyruvate synthase subunit PorC
2-oxoglutarate oxidoreductase subunit KorB
hypothetical protein
2-oxoglutarate oxidoreductase subunit KorA
Benzoyl-CoA oxygenase component A
Tetratricopeptide repeat protein
Exoenzyme S synthesis regulatory protein ExsA
Alpha/beta hydrolase family protein
Putative acetyltransferase
hypothetical protein
hypothetical protein
hypothetical protein
hypothetical protein
Nucleotidyltransferase domain protein
hypothetical protein
hypothetical protein
hypothetical protein
hypothetical protein
hypothetical protein
hypothetical protein
hypothetical protein
site-specific tyrosine recombinase XerD
hypothetical protein
putative AAA-ATPase
hypothetical protein
hypothetical protein
hypothetical protein
Alpha-xylosidase
Methionine synthase
Sodium/glucose cotransporter
Vitamin B12 dependent methionine synthase%2C activation domain
Uroporphyrinogen decarboxylase
Ribose-5-phosphate isomerase B
Transketolase
Intracellular exo-alpha-L-arabinofuranosidase 2
hypothetical protein
Xylulose kinase
L-arabinose isomerase
L-ribulose-5-phosphate 4-epimerase UlaF
bifunctional nicotinamide mononucleotide adenylyltransferase/ADP-ribose pyrophosphatase
Sodium/glucose cotransporter
Aldose 1-epimerase precursor
species
Bx
Bo

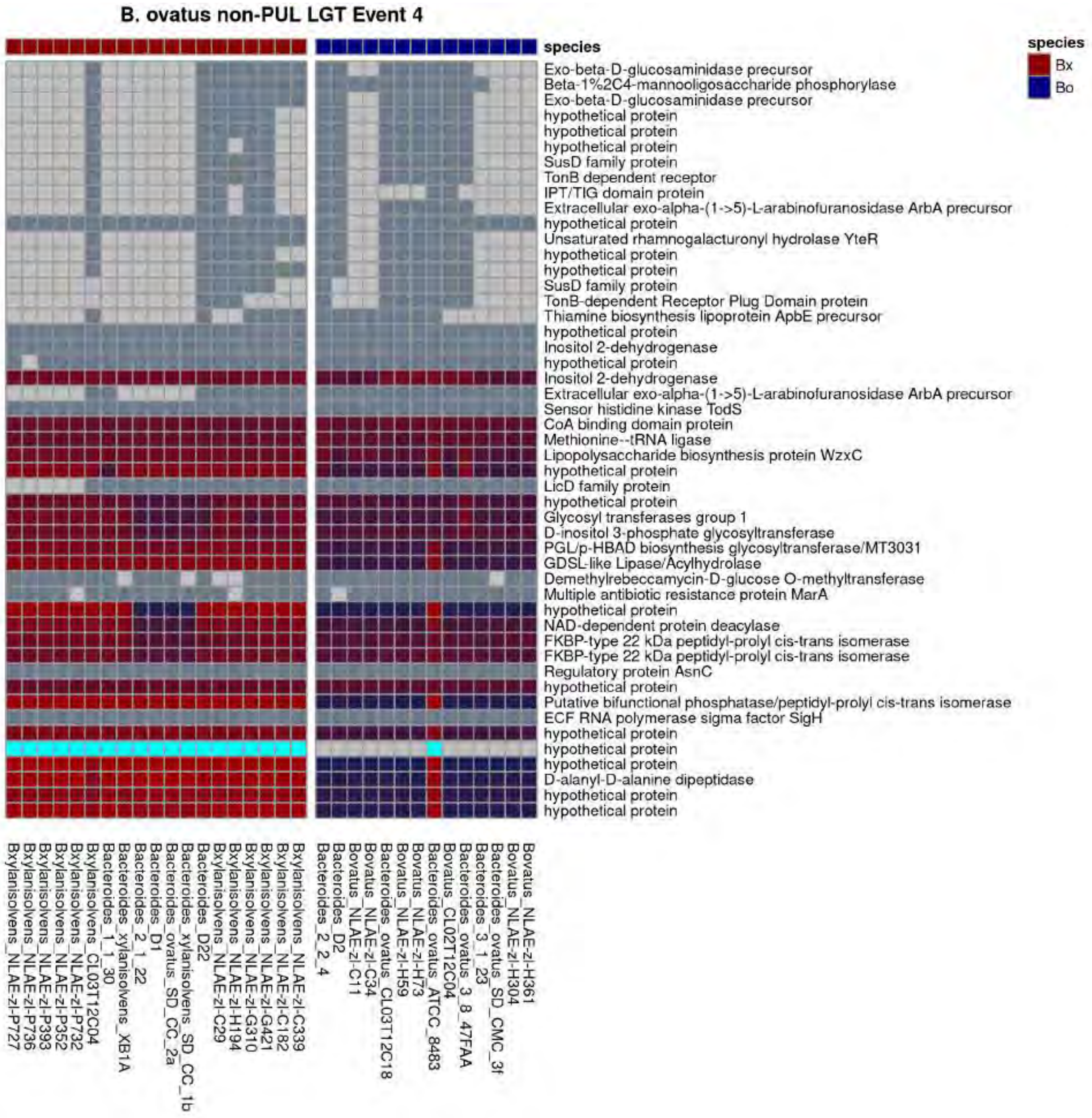
B. ovatus non-PUL LGT Event 4
species
Exo-beta-D-glucosaminidase precursor
Beta-1%2C4-mannooligosaccharide phosphorylase
Exo-beta-D-glucosaminidase precursor
hypothetical protein
hypothetical protein
hypothetical protein
SusD family protein
TonB dependent receptor
IPT/TIG domain protein
Extracellular exo-alpha-(1->5)-L-arabinofuranosidase ArbA precursor
hypothetical protein
Unsaturated rhamnogalacturonyl hydrolase YteR
hypothetical protein
hypothetical protein
SusD family protein
TonB-dependent Receptor Plug Domain protein
Thiamine biosynthesis lipoprotein ApbE precursor
hypothetical protein
Inositol 2-dehydrogenase
hypothetical protein
Inositol 2-dehydrogenase
Extracellular exo-alpha-(1->5)-L-arabinofuranosidase ArbA precursor
Sensor histidine kinase TodS
CoA binding domain protein
Methionine--tRNA ligase
Lipopolysaccharide biosynthesis protein WzxC
hypothetical protein
LicD family protein
hypothetical protein
Glycosyl transferases group 1
D-inositol 3-phosphate glycosyltransferase
PGL/p-HBAD biosynthesis glycosyltransferase/MT3031
GDSL-like Lipase/Acylhydrolase
Demethylrebeccamycin-D-glucose O-methyltransferase
Multiple antibiotic resistance protein MarA
hypothetical protein
NAD-dependent protein deacylase
FKBP-type 22 kDa peptidyl-prolyl cis-trans isomerase
FKBP-type 22 kDa peptidyl-prolyl cis-trans isomerase
Regulatory protein AsnC
hypothetical protein
Putative bifunctional phosphatase/peptidyl-prolyl cis-trans isomerase
ECF RNA polymerase sigma factor SigH
hypothetical protein
hypothetical protein
hypothetical protein
D-alanyl-D-alanine dipeptidase
hypothetical protein
hypothetical protein
species
Bx
Bo
Bxylanisolvens_NLAE-zl-P727
Bxylanisolvens_NLAE-zl-P736
Bxylanisolvens_NLAE-zl-P393
Bxylanisolvens_NLAE-zl-P352
Bxylanisolvens_NLAE-zl-P732
Bxylanisolvens_CL03T12C04
Bacteroides_1_1_30
Bacteroides_xylanisolvens_XB1A
Bacteroides_2_1_22
Bacteroides_D1
Bacteroides_ovatus_SD_CC_2a
Bacteroides_xylanisolvens_SD_CC_1b
Bacteroides_D22
Bxylanisolvens_NLAE-zl-C29
Bxylanisolvens_NLAE-zl-H194
Bxylanisolvens_NLAE-zl-G310
Bxylanisolvens_NLAE-zl-G421
Bxylanisolvens_NLAE-zl-C182
Bxylanisolvens_NLAE-zl-C339
Bacteroides_2_2_4
Bacteroides_D2
Bovatus_NLAE-zl-C11
Bovatus_NLAE-zl-C34
Bacteroides_ovatus_CL03T12C18
Bovatus_NLAE-zl-H59
Bovatus_NLAE-zl-H73
Bacteroides_ovatus_ATCC_8483
Bovatus_CL02T12C04
Bacteroides_ovatus_3_8_47FAA
Bacteroides_3_1_23
Bacteroides_ovatus_SD_CMC_3f
Bovatus_NLAE-zl-H304
Bovatus_NLAE-zl-H361

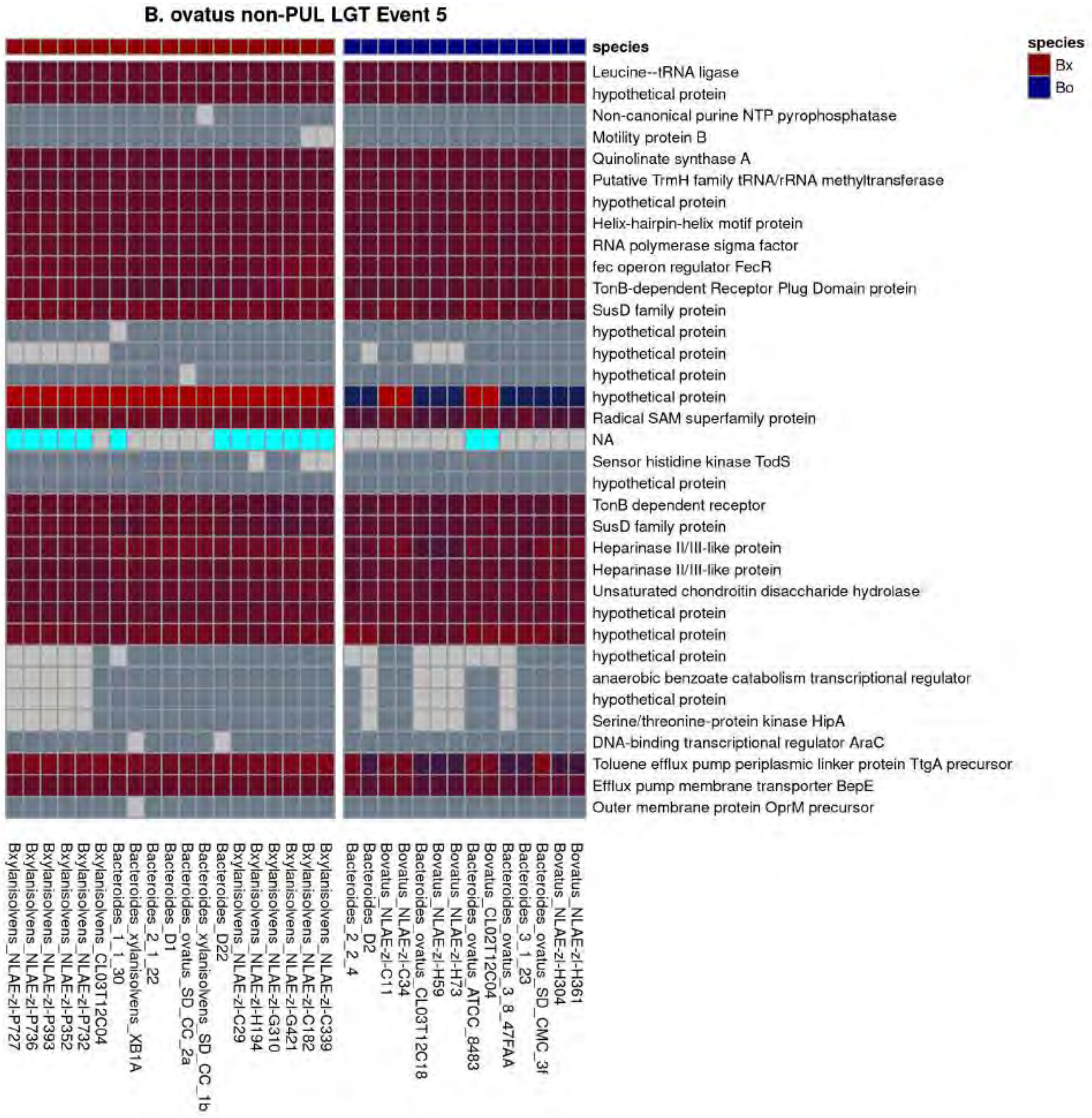
B. ovatus non-PUL LGT Event 5
species
Bx
Bo
Leucine--tRNA ligase
hypothetical protein
Non-canonical purine NTP pyrophosphatase
Motility protein B
Quinolinate synthase A
Putative TrmH family tRNA/rRNA methyltransferase
hypothetical protein
Helix-hairpin-helix motif protein
RNA polymerase sigma factor
fec operon regulator FecR
TonB-dependent Receptor Plug Domain protein
SusD family protein
hypothetical protein
hypothetical protein
hypothetical protein
hypothetical protein
Radical SAM superfamily protein
NA
Sensor histidine kinase TodS
hypothetical protein
TonB dependent receptor
SusD family protein
Heparinase II/III-like protein
Heparinase II/III-like protein
Unsaturated chondroitin disaccharide hydrolase
hypothetical protein
hypothetical protein
hypothetical protein
anaerobic benzoate catabolism transcriptional regulator
hypothetical protein
Serine/threonine-protein kinase HipA
DNA-binding transcriptional regulator AraC
Toluene efflux pump periplasmic linker protein TtgA precursor
Efflux pump membrane transporter BepE
Outer membrane protein OprM precursor
Bxylanisolvens_NLAE-zl-P727
Bxylanisolvens_NLAE-zl-P736
Bxylanisolvens_NLAE-zl-P393
Bxylanisolvens_NLAE-zl-P352
Bxylanisolvens_NLAE-zl-P732
Bxylanisolvens_CL03T12C04
Bacteroides_1_1_30
Bacteroides_xylanisolvens_XB1A
Bacteroides_2_1_22
Bacteroides_D1
Bacteroides_ovatus_SD_CC_2a
Bacteroides_xylanisolvens_SD_CC_1b
Bacteroides_D22
Bxylanisolvens_NLAE-zl-C29
Bxylanisolvens_NLAE-zl-H194
Bxylanisolvens_NLAE-zl-G310
Bxylanisolvens_NLAE-zl-G421
Bxylanisolvens_NLAE-zl-C182
Bxylanisolvens_NLAE-zl-C339
Bacteroides_2_2_4
Bacteroides_D2
Bovatus_NLAE-zl-C11
Bovatus_NLAE-zl-C34
Bacteroides_ovatus_CL03T12C18
Bovatus_NLAE-zl-H59
Bovatus_NLAE-zl-H73
Bacteroides_ovatus_ATCC_8483
Bovatus_CL02T12C04
Bacteroides_ovatus_3_8_47FAA
Bacteroides_3_1_23
Bacteroides_ovatus_SD_CMC_3f
Bovatus_NLAE-zl-H304
Bovatus_NLAE-zl-H361

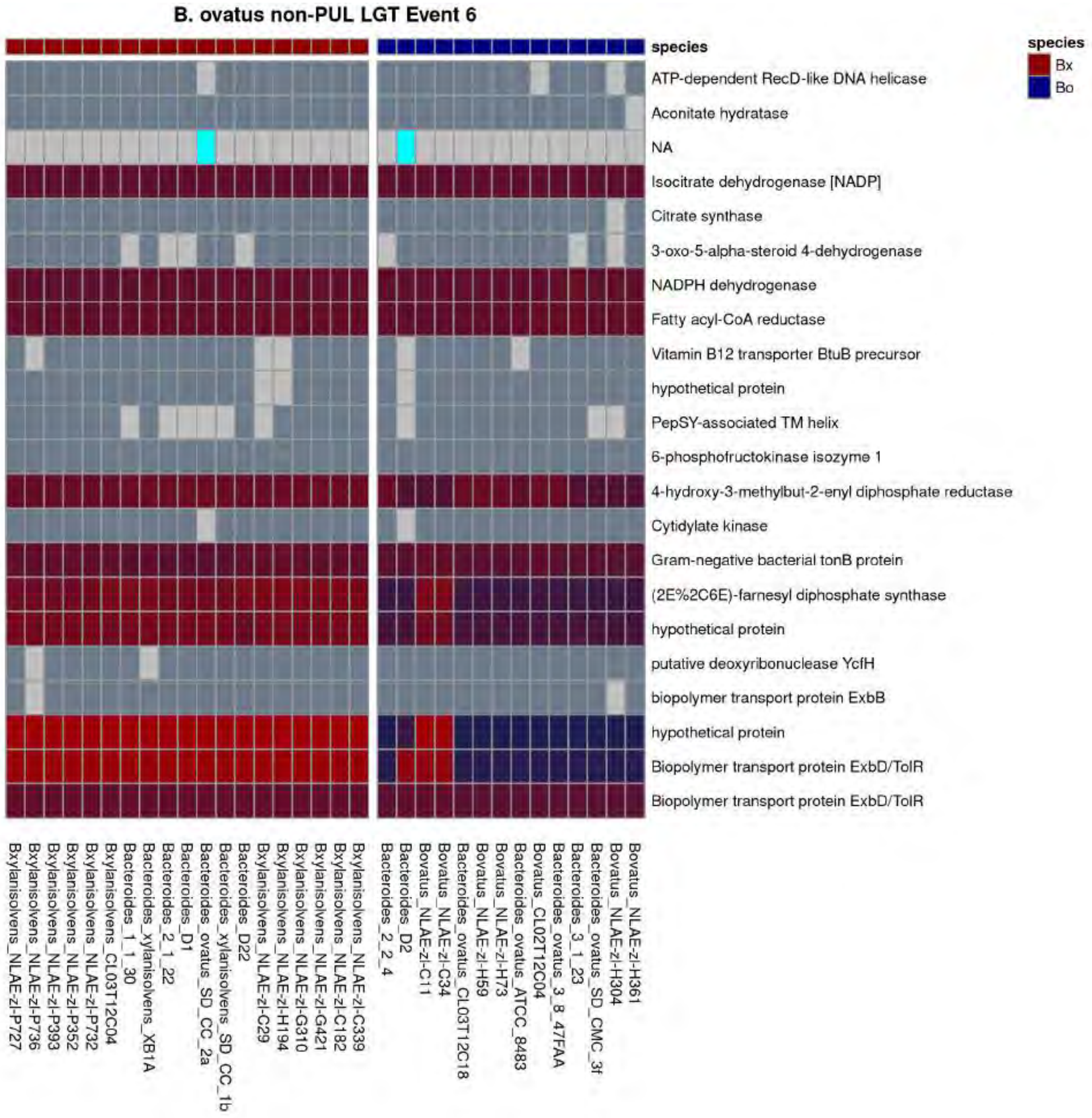
B. ovatus non-PUL LGT Event 6
species
ATP-dependent RecD-like DNA helicase
Aconitate hydratase
NA
Isocitrate dehydrogenase [NADP]
Citrate synthase
3-oxo-5-alpha-steroid 4-dehydrogenase
NADPH dehydrogenase
Fatty acyl-CoA reductase
Vitamin B12 transporter BtuB precursor
hypothetical protein
PepSY-associated TM helix
6-phosphofructokinase isozyme 1
4-hydroxy-3-methylbut-2-enyl diphosphate reductase
Cytidylate kinase
Gram-negative bacterial tonB protein
(2E%2C6E)-farnesyl diphosphate synthase
hypothetical protein
putative deoxyribonuclease YcfH
biopolymer transport protein ExbB
hypothetical protein
Biopolymer transport protein ExbD/TolR
Biopolymer transport protein ExbD/TolR
species
Bx
Bo
Bxylanisolvens_NLAE-zl-P727
Bxylanisolvens_NLAE-zl-P736
Bxylanisolvens_NLAE-zl-P393
Bxylanisolvens_NLAE-zl-P352
Bxylanisolvens_NLAE-zl-P732
Bxylanisolvens_CL03T12C04
Bacteroides_1_1_30
Bacteroides_xylanisolvens_XB1A
Bacteroides_2_1_22
Bacteroides_D1
Bacteroides_ovatus_SD_CC_2a
Bacteroides_xylanisolvens_SD_CC_1b
Bacteroides_D22
Bxylanisolvens_NLAE-zl-C29
Bxylanisolvens_NLAE-zl-H194
Bxylanisolvens_NLAE-zl-G310
Bxylanisolvens_NLAE-zl-G421
Bxylanisolvens_NLAE-zl-C182
Bxylanisolvens_NLAE-zl-C339
Bacteroides_2_2_4
Bacteroides_D2
Bovatus_NLAE-zl-C11
Bovatus_NLAE-zl-C34
Bacteroides_ovatus_CL03T12C18
Bovatus_NLAE-zl-H59
Bovatus_NLAE-zl-H73
Bacteroides_ovatus_ATCC_8483
Bovatus_CL02T12C04
Bacteroides_ovatus_3_8_47FAA
Bacteroides_3_1_23
Bacteroides_ovatus_SD_CMC_3f
Bovatus_NLAE-zl-H304
Bovatus_NLAE-zl-H361

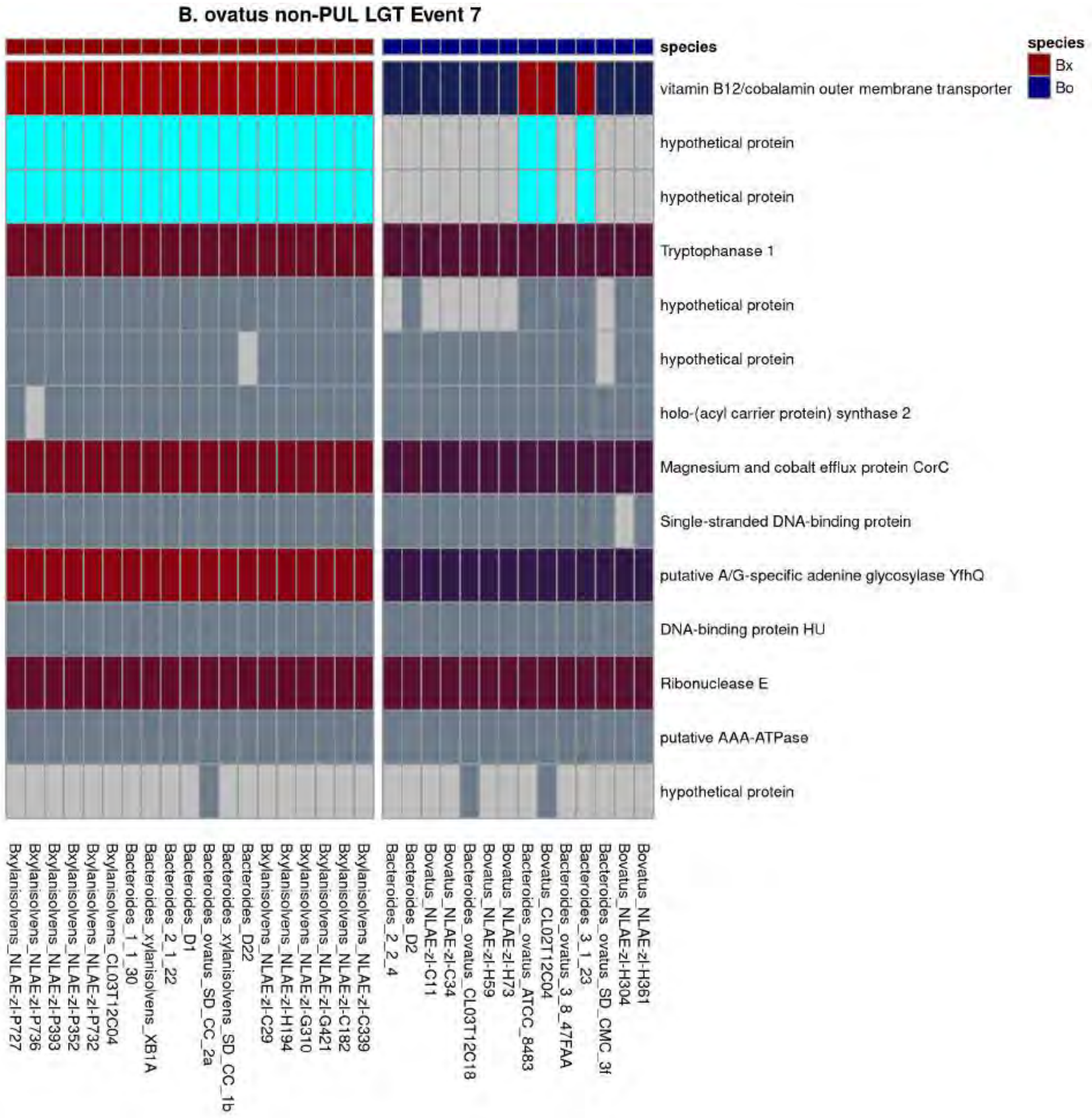
B. ovatus non-PUL LGT Event 7
species
vitamin B12/cobalamin outer membrane transporter
hypothetical protein
hypothetical protein
Tryptophanase 1
hypothetical protein
hypothetical protein
holo-(acyl carrier protein) synthase 2
Magnesium and cobalt efflux protein CorC
Single-stranded DNA-binding protein
putative A/G-specific adenine glycosylase YfhQ
DNA-binding protein HU
Ribonuclease E
putative AAA-ATPase
hypothetical protein
species
Bx
Bo
Bxylanisolvens_NLAE-zl-P727
Bxylanisolvens_NLAE-zl-P736
Bxylanisolvens_NLAE-zl-P393
Bxylanisolvens_NLAE-zl-P352
Bxylanisolvens_NLAE-zl-P732
Bxylanisolvens_CL03T12C04
Bacteroides_1_1_30
Bacteroides_xylanisolvens_XB1A
Bacteroides_2_1_22
Bacteroides_D1
Bacteroides_ovatus_SD_CC_2a
Bacteroides_xylanisolvens_SD_CC_1b
Bacteroides_D22
Bxylanisolvens_NLAE-zl-C29
Bxylanisolvens_NLAE-zl-H194
Bxylanisolvens_NLAE-zl-G310
Bxylanisolvens_NLAE-zl-G421
Bxylanisolvens_NLAE-zl-C182
Bxylanisolvens_NLAE-zl-C339
Bacteroides_2_2_4
Bacteroides_D2
Bovatus_NLAE-zl-C11
Bovatus_NLAE-zl-C34
Bacteroides_ovatus_CL03T12C18
Bovatus_NLAE-zl-H59
Bovatus_NLAE-zl-H73
Bacteroides_ovatus_ATCC_8483
Bovatus_CL02T12C04
Bacteroides_ovatus_3_8_47FAA
Bacteroides_3_1_23
Bacteroides_ovatus_SD_CMC_3f
Bovatus_NLAE-zl-H304
Bovatus_NLAE-zl-H361

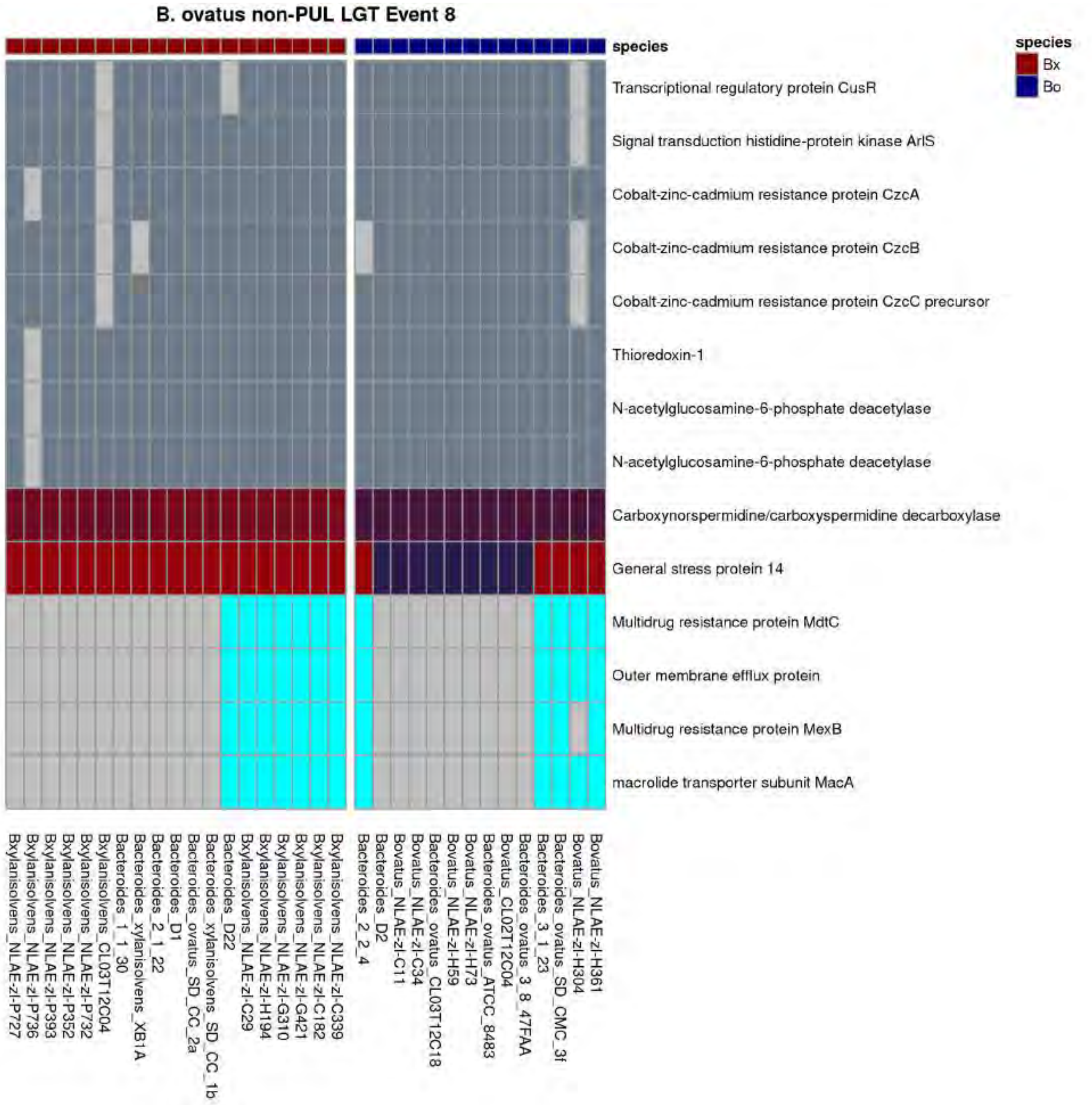

B. ovatus non-PUL LGT Event 8
species
Bx
Bo
Transcriptional regulatory protein CusR
Signal transduction histidine-protein kinase ArlS
Cobalt-zinc-cadmium resistance protein CzcA
Cobalt-zinc-cadmium resistance protein CzcB
Cobalt-zinc-cadmium resistance protein CzcC precursor
Thioredoxin-1
N-acetylglucosamine-6-phosphate deacetylase
N-acetylglucosamine-6-phosphate deacetylase
Carboxynorspermidine/carboxyspermidine decarboxylase
General stress protein 14
Multidrug resistance protein MdtC
Outer membrane efflux protein
Multidrug resistance protein MexB
macrolide transporter subunit MacA
Bxylanisolvens_NLAE-zl-P727
Bxylanisolvens_NLAE-zl-P736
Bxylanisolvens_NLAE-zl-P393
Bxylanisolvens_NLAE-zl-P352
Bxylanisolvens_NLAE-zl-P732
Bxylanisolvens_CL03T12C04
Bacteroides_1_1_30
Bacteroides_xylanisolvens_XB1A
Bacteroides_2_1_22
Bacteroides_D1
Bacteroides_ovatus_SD_CC_2a
Bacteroides_xylanisolvens_SD_CC_1b
Bacteroides_D22
Bxylanisolvens_NLAE-zl-C29
Bxylanisolvens_NLAE-zl-H194
Bxylanisolvens_NLAE-zl-G310
Bxylanisolvens_NLAE-zl-G421
Bxylanisolvens_NLAE-zl-C182
Bxylanisolvens_NLAE-zl-C339
Bacteroides_2_2_4
Bacteroides_D2
Bovatus_NLAE-zl-C11
Bovatus_NLAE-zl-C34
Bacteroides_ovatus_CL03T12C18
Bovatus_NLAE-zl-H59
Bovatus_NLAE-zl-H73
Bacteroides_ovatus_ATCC_8483
Bovatus_CL02T12C04
Bacteroides_ovatus_3_8_47FAA
Bacteroides_3_1_23
Bacteroides_ovatus_SD_CMC_3f
Bovatus_NLAE-zl-H304
Bovatus_NLAE-zl-H361

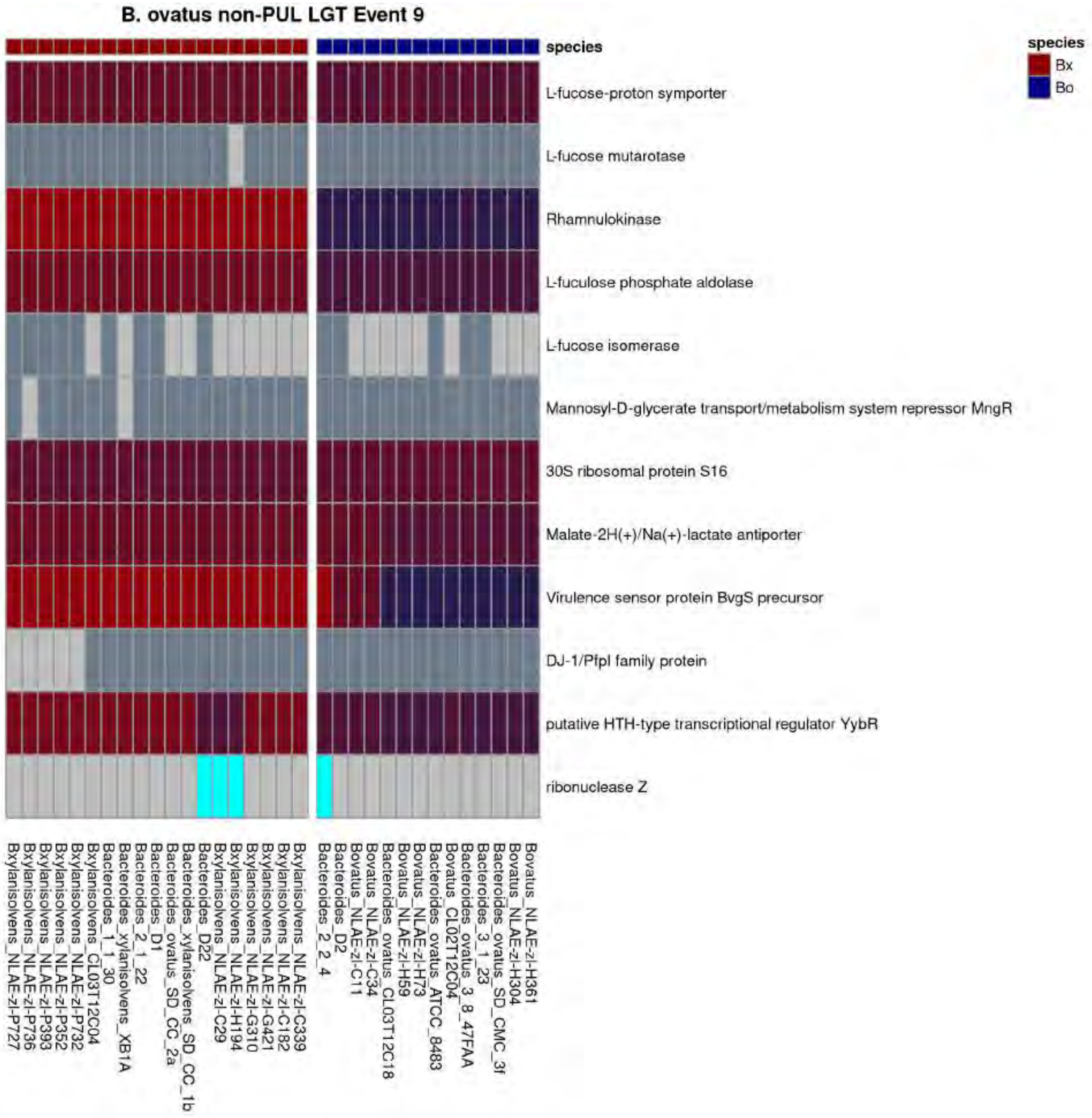
B. ovatus non-PUL LGT Event 9
species
Bx
Bo
species
L-fucose-proton symporter
L-fucose mutarotase
Rhamnulokinase
L-fuculose phosphate aldolase
L-fucose isomerase
Mannosyl-D-glycerate transport/metabolism system repressor MngR
30S ribosomal protein S16
Malate-2H(+)/Na(+)-lactate antiporter
Virulence sensor protein BvgS precursor
DJ-1/PfpI family protein
putative HTH-type transcriptional regulator YybR
ribonuclease Z
Bxylanisolvens_NLAE-zl-P727
Bxylanisolvens_NLAE-zl-P736
Bxylanisolvens_NLAE-zl-P393
Bxylanisolvens_NLAE-zl-P352
Bxylanisolvens_NLAE-zl-P732
Bxylanisolvens_CL03T12C04
Bacteroides_1_1_30
Bacteroides_xylanisolvens_XB1A
Bacteroides_2_1_22
Bacteroides_D1
Bacteroides_ovatus_SD_CC_2a
Bacteroides_xylanisolvens_SD_CC_1b
Bacteroides_D22
Bxylanisolvens_NLAE-zl-C29
Bxylanisolvens_NLAE-zl-H194
Bxylanisolvens_NLAE-zl-G310
Bxylanisolvens_NLAE-zl-G421
Bxylanisolvens_NLAE-zl-C182
Bxylanisolvens_NLAE-zl-C339
Bacteroides_2_2_4
Bacteroides_D2
Bovatus_NLAE-zl-C11
Bovatus_NLAE-zl-C34
Bacteroides_ovatus_CL03T12C18
Bovatus_NLAE-zl-H59
Bovatus_NLAE-zl-H73
Bacteroides_ovatus_ATCC_8483
Bovatus_CL02T12C04
Bacteroides_ovatus_3_8_47FAA
Bacteroides_3_1_23
Bacteroides_ovatus_SD_CMC_3f
Bovatus_NLAE-zl-H304
Bovatus_NLAE-zl-H361

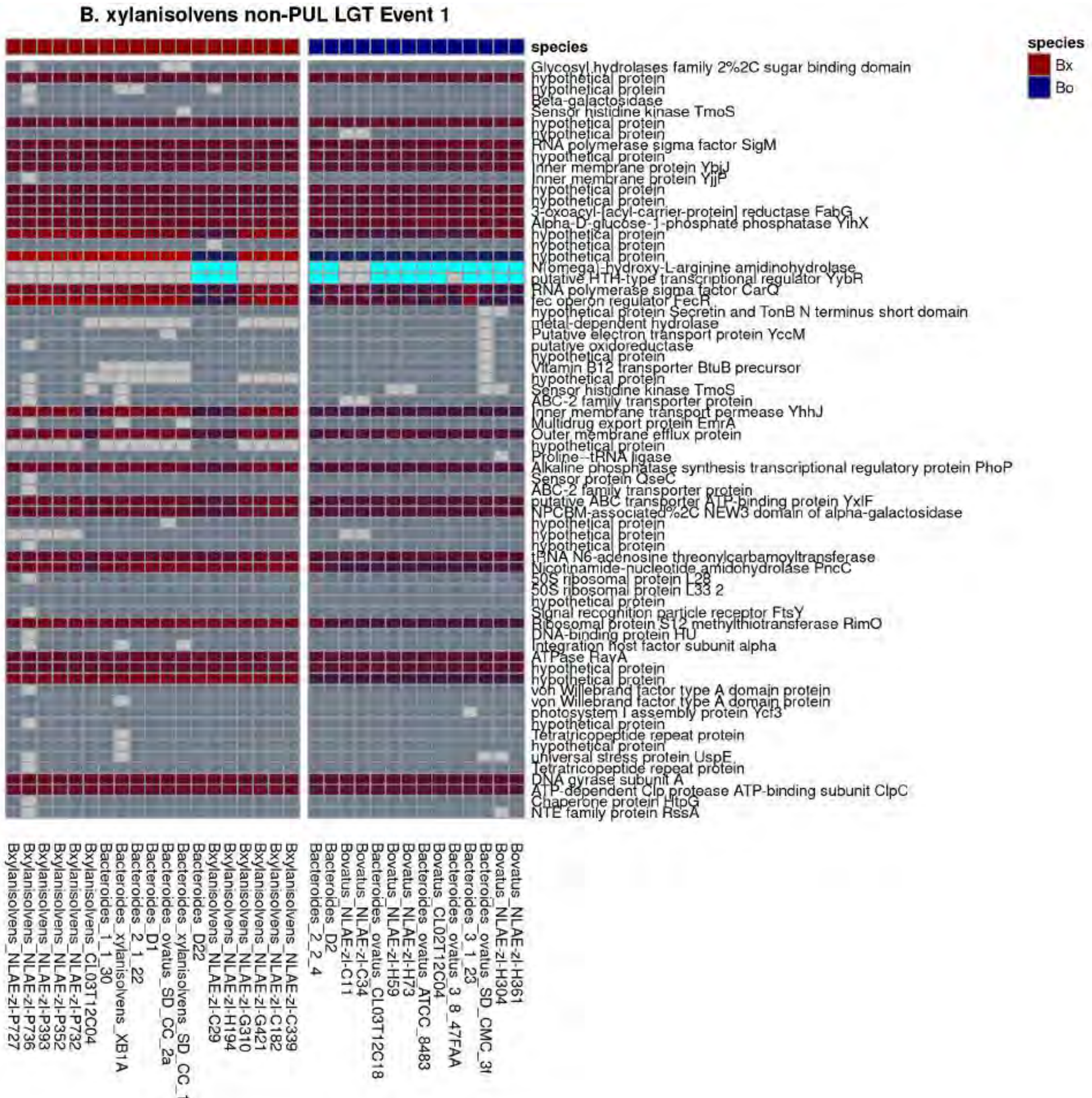
B. xylanisolvens non-PUL LGT Event 1
species
Bx
Bo
Glycosyl hydrolases family 2%2C sugar binding domain
hypothetical protein
hypothetical protein
Beta-galactosidase
Sensor histidine kinase TmoS
hypothetical protein
hypothetical protein
RNA polymerase sigma factor SigM
hypothetical protein
Inner membrane protein YbjJ
Inner membrane protein YjjP
hypothetical protein
hypothetical protein
3-oxoacyl-[acyl-carrier-protein] reductase FabG
Alpha-D-glucose-1-phosphate phosphatase YihX
hypothetical protein
hypothetical protein
hypothetical protein
N(omega)-hydroxy-L-arginine amidinohydrolase
putative HTH-type transcriptional regulator YybR
RNA polymerase sigma factor CarQ
fec operon regulator FecR
hypothetical protein Secretin and TonB N terminus short domain
metal-dependent hydrolase
Putative electron transport protein YccM
putative oxidoreductase
hypothetical protein
Vitamin B12 transporter BtuB precursor
hypothetical protein
Sensor histidine kinase TmoS
ABC-2 family transporter protein
Inner membrane transport permease YhhJ
Multidrug export protein EmrA
Outer membrane efflux protein
hypothetical protein
Proline--tRNA ligase
Alkaline phosphatase synthesis transcriptional regulatory protein PhoP
Sensor protein QseC
ABC-2 family transporter protein
putative ABC transporter ATP-binding protein YxlF
NPCBM-associated%2C NEW3 domain of alpha-galactosidase
hypothetical protein
hypothetical protein
hypothetical protein
tRNA N6-adenosine threonylcarbamoyltransferase
Nicotinamide-nucleotide amidohydrolase PncC
50S ribosomal protein L28
50S ribosomal protein L33 2
hypothetical protein
Signal recognition particle receptor FtsY
Ribosomal protein S12 methylthiotransferase RimO
DNA-binding protein HU
Integration host factor subunit alpha
ATPase RavA
hypothetical protein
hypothetical protein
von Willebrand factor type A domain protein
von Willebrand factor type A domain protein
photosystem I assembly protein Ycf3
hypothetical protein
Tetratricopeptide repeat protein
hypothetical protein
universal stress protein UspE
Tetratricopeptide repeat protein
DNA gyrase subunit A
ATP-dependent Clp protease ATP-binding subunit ClpC
Chaperone protein HtpG
NTE family protein RssA
Bxylanisolvens_NLAE-zl-P727
Bxylanisolvens_NLAE-zl-P736
Bxylanisolvens_NLAE-zl-P393
Bxylanisolvens_NLAE-zl-P352
Bxylanisolvens_NLAE-zl-P732
Bxylanisolvens_CL03T12C04
Bacteroides_1_1_30
Bacteroides_xylanisolvens_XB1A
Bacteroides_2_1_22
Bacteroides_D1
Bacteroides_ovatus_SD_CC_2a
Bacteroides_xylanisolvens_SD_CC_1b
Bacteroides_D22
Bxylanisolvens_NLAE-zl-C29
Bxylanisolvens_NLAE-zl-H194
Bxylanisolvens_NLAE-zl-G310
Bxylanisolvens_NLAE-zl-G421
Bxylanisolvens_NLAE-zl-C182
Bxylanisolvens_NLAE-zl-C339
Bacteroides_2_2_4
Bacteroides_D2
Bovatus_NLAE-zl-C11
Bovatus_NLAE-zl-C34
Bacteroides_ovatus_CL03T12C18
Bovatus_NLAE-zl-H59
Bovatus_NLAE-zl-H73
Bacteroides_ovatus_ATCC_8483
Bovatus_CL02T12C04
Bacteroides_ovatus_3_8_47FAA
Bacteroides_3_1_23
Bacteroides_ovatus_SD_CMC_3f
Bovatus_NLAE-zl-H304
Bovatus_NLAE-zl-H361

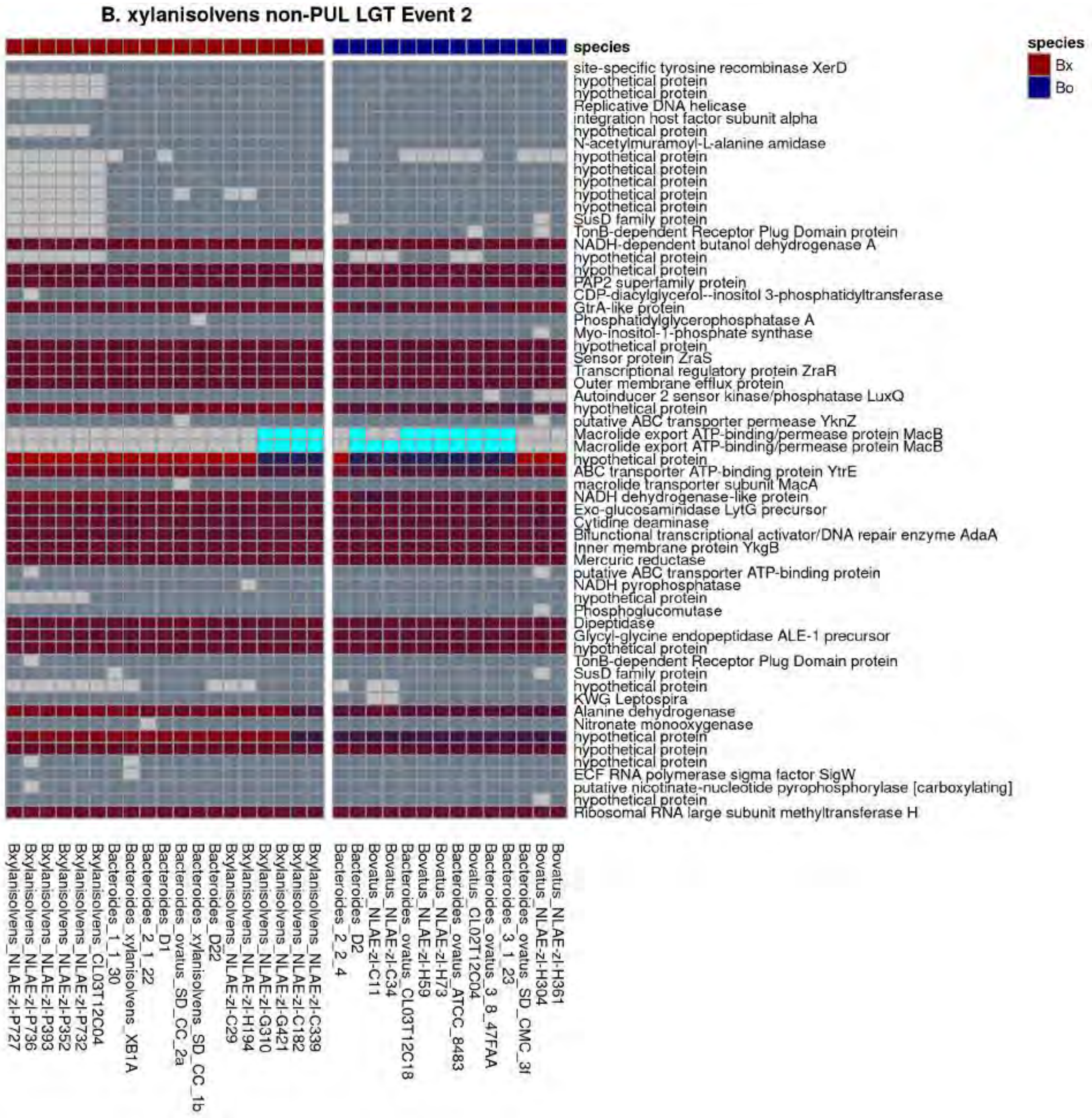
B. xylanisolvens non-PUL LGT Event 2
species
Bx
Bo
site-specific tyrosine recombinase XerD
hypothetical protein
hypothetical protein
Replicative DNA helicase
integration host factor subunit alpha
hypothetical protein
N-acetylmuramoyl-L-alanine amidase
hypothetical protein
hypothetical protein
hypothetical protein
hypothetical protein
hypothetical protein
SusD family protein
TonB-dependent Receptor Plug Domain protein
NADH-dependent butanol dehydrogenase A
hypothetical protein
hypothetical protein
PAP2 superfamily protein
CDP-diacylglycerol--inositol 3-phosphatidyltransferase
GtrA-like protein
Phosphatidylglycerophosphatase A
Myo-inositol-1-phosphate synthase
hypothetical protein
Sensor protein ZraS
Transcriptional regulatory protein ZraR
Outer membrane efflux protein
Autoinducer 2 sensor kinase/phosphatase LuxQ
hypothetical protein
putative ABC transporter permease YknZ
Macrolide export ATP-binding/permease protein MacB
Macrolide export ATP-binding/permease protein MacB
hypothetical protein
ABC transporter ATP-binding protein YtrE
macrolide transporter subunit MacA
NADH dehydrogenase-like protein
Exo-glucosaminidase LytG precursor
Cytidine deaminase
Bifunctional transcriptional activator/DNA repair enzyme AdaA
Inner membrane protein YkgB
Mercuric reductase
putative ABC transporter ATP-binding protein
NADH pyrophosphatase
hypothetical protein
Phosphoglucomutase
Dipeptidase
Glycyl-glycine endopeptidase ALE-1 precursor
hypothetical protein
TonB-dependent Receptor Plug Domain protein
SusD family protein
hypothetical protein
KWG Leptospira
Alanine dehydrogenase
Nitronate monooxygenase
hypothetical protein
hypothetical protein
hypothetical protein
ECF RNA polymerase sigma factor SigW
putative nicotinate-nucleotide pyrophosphorylase [carboxylating]
hypothetical protein
Ribosomal RNA large subunit methyltransferase H
Bxylanisolvens_NLAE-zl-P727
Bxylanisolvens_NLAE-zl-P736
Bxylanisolvens_NLAE-zl-P393
Bxylanisolvens_NLAE-zl-P352
Bxylanisolvens_NLAE-zl-P732
Bxylanisolvens_CL03T12C04
Bacteroides_1_1_30
Bacteroides_xylanisolvens_XB1A
Bacteroides_2_1_22
Bacteroides_D1
Bacteroides_ovatus_SD_CC_2a
Bacteroides_xylanisolvens_SD_CC_1b
Bacteroides_D22
Bxylanisolvens_NLAE-zl-C29
Bxylanisolvens_NLAE-zl-H194
Bxylanisolvens_NLAE-zl-G310
Bxylanisolvens_NLAE-zl-G421
Bxylanisolvens_NLAE-zl-C182
Bxylanisolvens_NLAE-zl-C339
Bacteroides_2_2_4
Bacteroides_D2
Bovatus_NLAE-zl-C11
Bovatus_NLAE-zl-C34
Bacteroides_ovatus_CL03T12C18
Bovatus_NLAE-zl-H59
Bovatus_NLAE-zl-H73
Bacteroides_ovatus_ATCC_8483
Bovatus_CL02T12C04
Bacteroides_ovatus_3_8_47FAA
Bacteroides_3_1_23
Bacteroides_ovatus_SD_CMC_3f
Bovatus_NLAE-zl-H304
Bovatus_NLAE-zl-H361

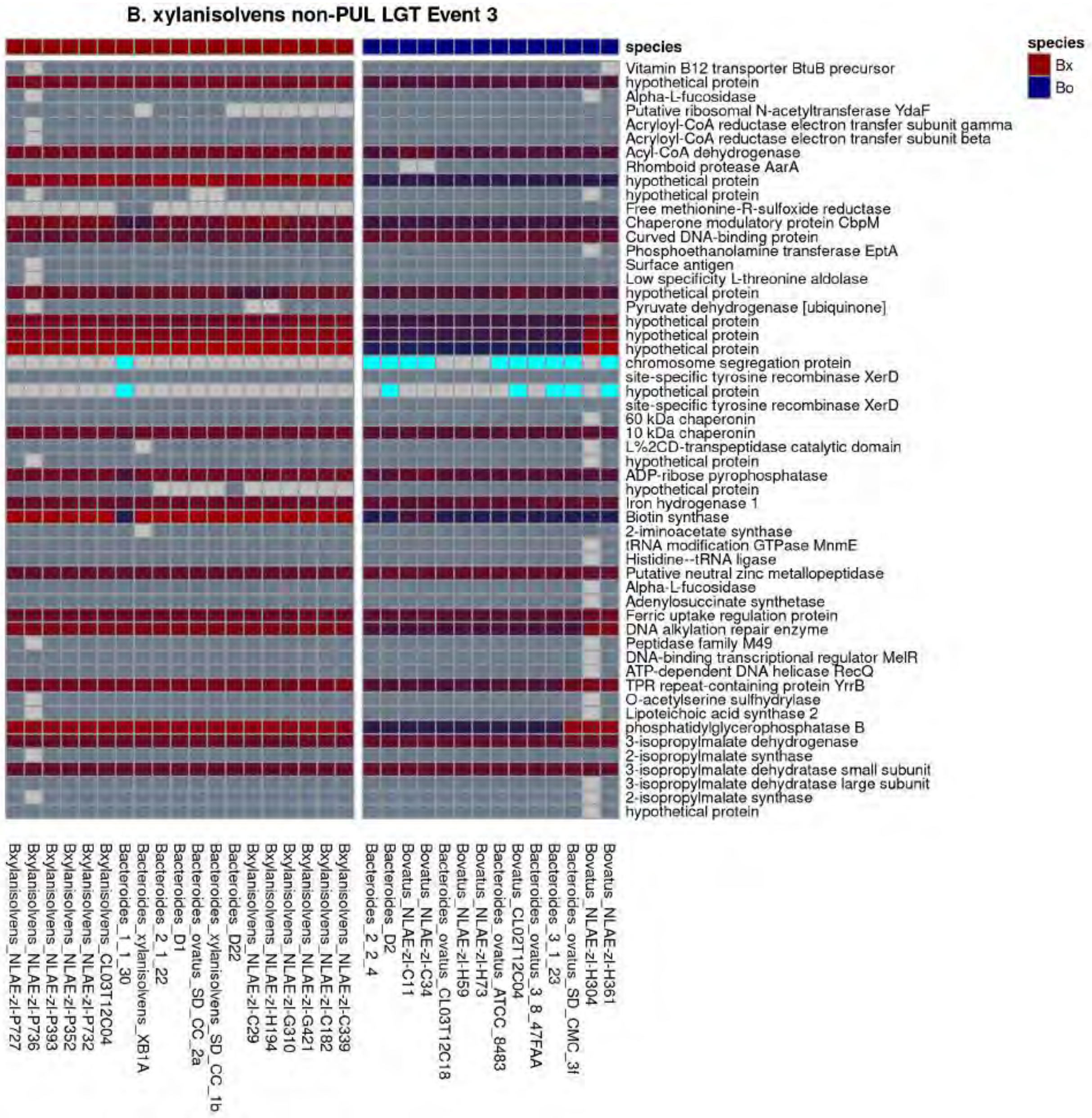
B. xylanisolvens non-PUL LGT Event 3
species
Bx
Bo
Vitamin B12 transporter BtuB precursor
hypothetical protein
Alpha-L-fucosidase
Putative ribosomal N-acetyltransferase YdaF
Acryloyl-CoA reductase electron transfer subunit gamma
Acryloyl-CoA reductase electron transfer subunit beta
Acyl-CoA dehydrogenase
Rhomboid protease AarA
hypothetical protein
hypothetical protein
Free methionine-R-sulfoxide reductase
Chaperone modulatory protein CbpM
Curved DNA-binding protein
Phosphoethanolamine transferase EptA
Surface antigen
Low specificity L-threonine aldolase
hypothetical protein
Pyruvate dehydrogenase [ubiquinone]
hypothetical protein
hypothetical protein
hypothetical protein
chromosome segregation protein
site-specific tyrosine recombinase XerD
hypothetical protein
site-specific tyrosine recombinase XerD
60 kDa chaperonin
10 kDa chaperonin
L%2CD-transpeptidase catalytic domain
hypothetical protein
ADP-ribose pyrophosphatase
hypothetical protein
Iron hydrogenase 1
Biotin synthase
2-iminoacetate synthase
tRNA modification GTPase MnmE
Histidine--tRNA ligase
Putative neutral zinc metallopeptidase
Alpha-L-fucosidase
Adenylosuccinate synthetase
Ferric uptake regulation protein
DNA alkylation repair enzyme
Peptidase family M49
DNA-binding transcriptional regulator MelR
ATP-dependent DNA helicase RecQ
TPR repeat-containing protein YrrB
O-acetylserine sulfhydrylase
Lipoteichoic acid synthase 2
phosphatidylglycerophosphatase B
3-isopropylmalate dehydrogenase
2-isopropylmalate synthase
3-isopropylmalate dehydratase small subunit
3-isopropylmalate dehydratase large subunit
2-isopropylmalate synthase
hypothetical protein
Bxylanisolvens_NLAE-zl-P727
Bxylanisolvens_NLAE-zl-P736
Bxylanisolvens_NLAE-zl-P393
Bxylanisolvens_NLAE-zl-P352
Bxylanisolvens_NLAE-zl-P732
Bxylanisolvens_CL03T12C04
Bacteroides_1_1_30
Bacteroides_xylanisolvens_XB1A
Bacteroides_2_1_22
Bacteroides_D1
Bacteroides_ovatus_SD_CC_2a
Bacteroides_xylanisolvens_SD_CC_1b
Bacteroides_D22
Bxylanisolvens_NLAE-zl-C29
Bxylanisolvens_NLAE-zl-H194
Bxylanisolvens_NLAE-zl-G310
Bxylanisolvens_NLAE-zl-G421
Bxylanisolvens_NLAE-zl-C182
Bxylanisolvens_NLAE-zl-C339
Bacteroides_2_2_4
Bacteroides_D2
Bovatus_NLAE-zl-C11
Bovatus_NLAE-zl-C34
Bacteroides_ovatus_CL03T12C18
Bovatus_NLAE-zl-H59
Bovatus_NLAE-zl-H73
Bacteroides_ovatus_ATCC_8483
Bovatus_CL02T12C04
Bacteroides_ovatus_3_8_47FAA
Bacteroides_3_1_23
Bacteroides_ovatus_SD_CMC_3f
Bovatus_NLAE-zl-H304
Bovatus_NLAE-zl-H361

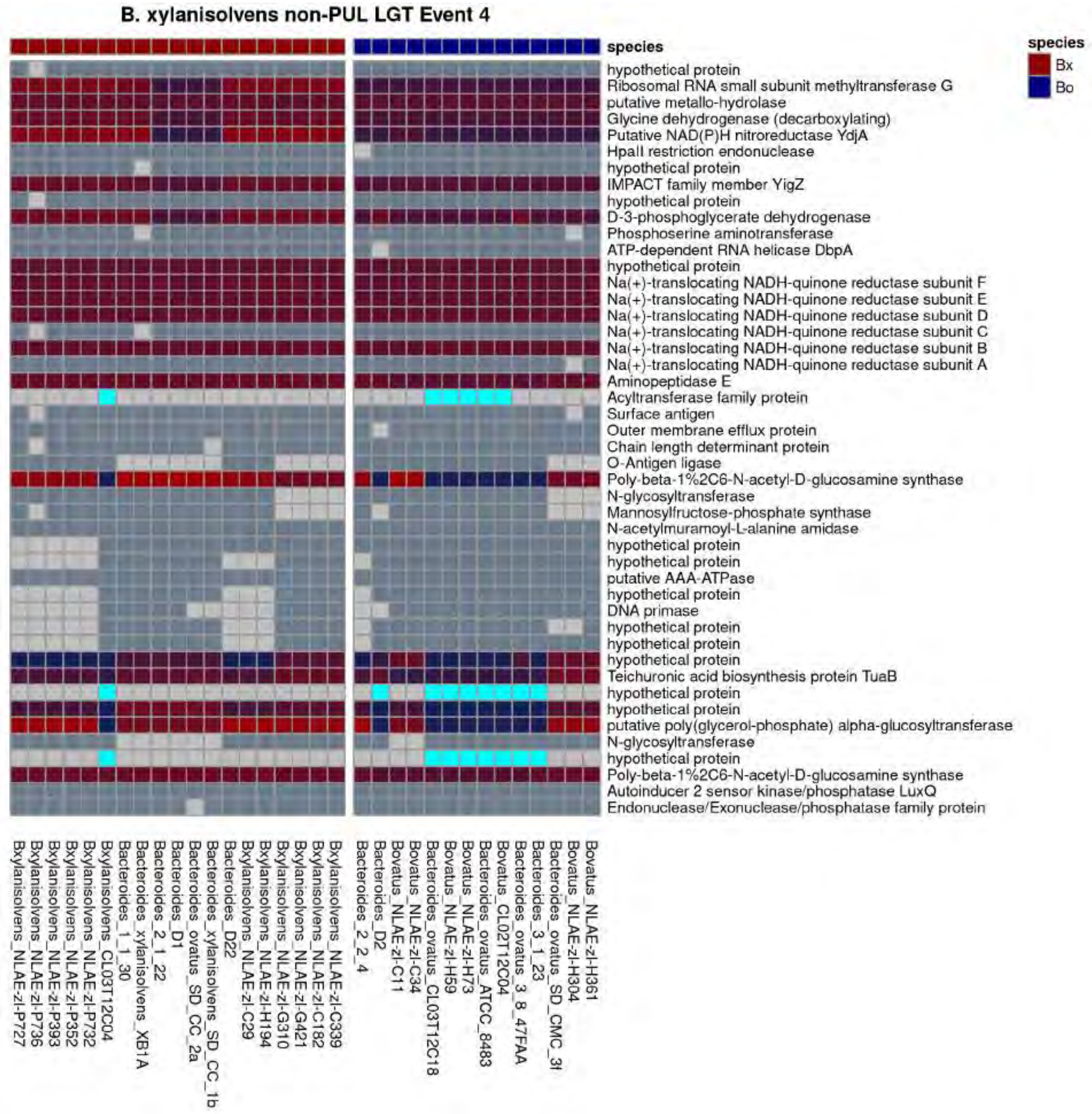
B. xylanisolvens non-PUL LGT Event 4
species
species
Bx
Bo
hypothetical protein
Ribosomal RNA small subunit methyltransferase G
putative metallo-hydrolase
Glycine dehydrogenase (decarboxylating)
Putative NAD(P)H nitroreductase YdjA
HpaII restriction endonuclease
hypothetical protein
IMPACT family member YigZ
hypothetical protein
D-3-phosphoglycerate dehydrogenase
Phosphoserine aminotransferase
ATP-dependent RNA helicase DbpA
hypothetical protein
Na(+)-translocating NADH-quinone reductase subunit F
Na(+)-translocating NADH-quinone reductase subunit E
Na(+)-translocating NADH-quinone reductase subunit D
Na(+)-translocating NADH-quinone reductase subunit C
Na(+)-translocating NADH-quinone reductase subunit B
Na(+)-translocating NADH-quinone reductase subunit A
Aminopeptidase E
Acyltransferase family protein
Surface antigen
Outer membrane efflux protein
Chain length determinant protein
O-Antigen ligase
Poly-beta-1%2C6-N-acetyl-D-glucosamine synthase
N-glycosyltransferase
Mannosylfructose-phosphate synthase
N-acetylmuramoyl-L-alanine amidase
hypothetical protein
hypothetical protein
putative AAA-ATPase
hypothetical protein
DNA primase
hypothetical protein
hypothetical protein
hypothetical protein
Teichuronic acid biosynthesis protein TuaB
hypothetical protein
hypothetical protein
putative poly(glycerol-phosphate) alpha-glucosyltransferase
N-glycosyltransferase
hypothetical protein
Poly-beta-1%2C6-N-acetyl-D-glucosamine synthase
Autoinducer 2 sensor kinase/phosphatase LuxQ
Endonuclease/Exonuclease/phosphatase family protein
Bxylanisolvens_NLAE-zl-P727
Bxylanisolvens_NLAE-zl-P736
Bxylanisolvens_NLAE-zl-P393
Bxylanisolvens_NLAE-zl-P352
Bxylanisolvens_NLAE-zl-P732
Bxylanisolvens_CL03T12C04
Bacteroides_xylanisolvens_XB1A
Bacteroides_1_1_30
Bacteroides_2_1_22
Bacteroides_D1
Bacteroides_ovatus_SD_CC_2a
Bacteroides_xylanisolvens_SD_CC_1b
Bacteroides_D22
Bxylanisolvens_NLAE-zl-C29
Bxylanisolvens_NLAE-zl-H194
Bxylanisolvens_NLAE-zl-G310
Bxylanisolvens_NLAE-zl-G421
Bxylanisolvens_NLAE-zl-C182
Bxylanisolvens_NLAE-zl-C339
Bacteroides_2_2_4
Bacteroides_D2
Bovatus_NLAE-zl-C11
Bovatus_NLAE-zl-C34
Bacteroides_ovatus_CL03T12C18
Bovatus_NLAE-zl-H59
Bovatus_NLAE-zl-H73
Bacteroides_ovatus_ATCC_8483
Bovatus_CL02T12C04
Bacteroides_ovatus_3_8_47FAA
Bacteroides_3_1_23
Bacteroides_ovatus_SD_CMC_3f
Bovatus_NLAE-zl-H304
Bovatus_NLAE-zl-H361

B. xylanisolvens non-PUL LGT Event 5

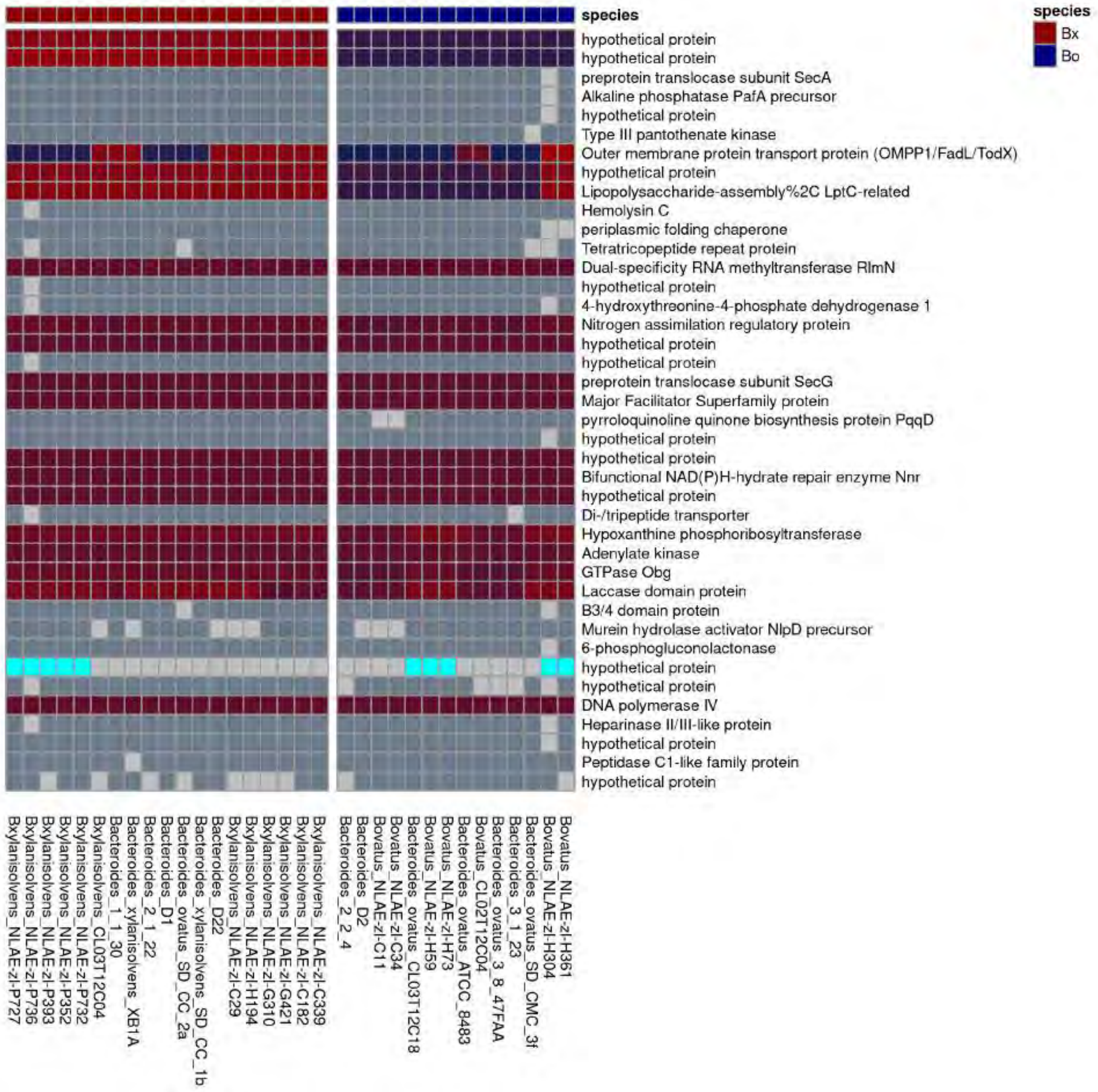

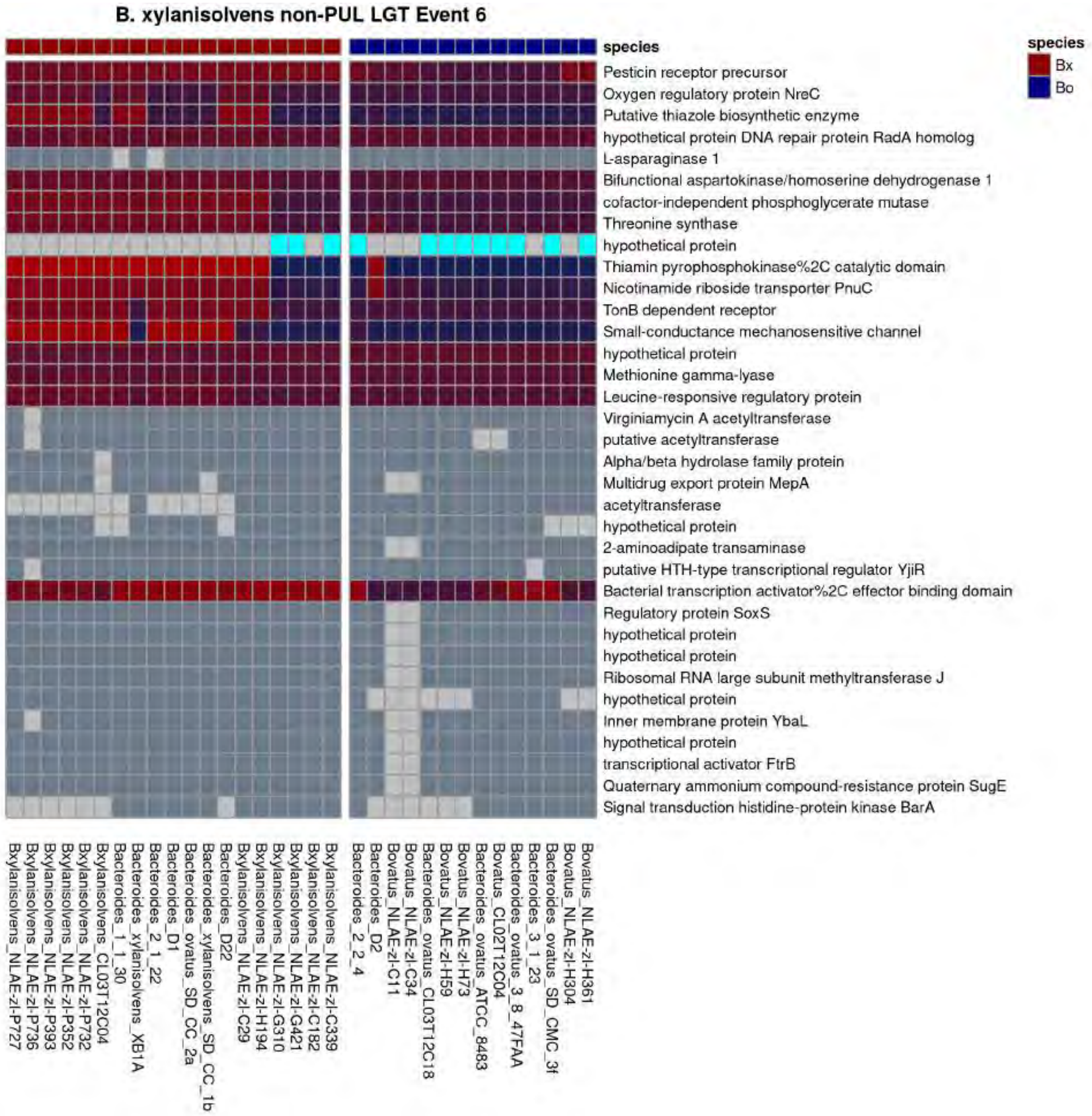
B. xylanisolvens non-PUL LGT Event 6
species
Pesticin receptor precursor
Oxygen regulatory protein NreC
Putative thiazole biosynthetic enzyme
hypothetical protein DNA repair protein RadA homolog
L-asparaginase 1
Bifunctional aspartokinase/homoserine dehydrogenase 1
cofactor-independent phosphoglycerate mutase
Threonine synthase
hypothetical protein
Thiamin pyrophosphokinase%2C catalytic domain
Nicotinamide riboside transporter PnuC
TonB dependent receptor
Small-conductance mechanosensitive channel
hypothetical protein
Methionine gamma-lyase
Leucine-responsive regulatory protein
Virginiamycin A acetyltransferase
putative acetyltransferase
Alpha/beta hydrolase family protein
Multidrug export protein MepA
acetyltransferase
hypothetical protein
2-aminoadipate transaminase
putative HTH-type transcriptional regulator YjiR
Bacterial transcription activator%2C effector binding domain
Regulatory protein SoxS
hypothetical protein
hypothetical protein
Ribosomal RNA large subunit methyltransferase J
hypothetical protein
Inner membrane protein YbaL
hypothetical protein
transcriptional activator FtrB
Quaternary ammonium compound-resistance protein SugE
Signal transduction histidine-protein kinase BarA
species
Bx
Bo
Bxylanisolvens_NLAE-zl-P727
Bxylanisolvens_NLAE-zl-P736
Bxylanisolvens_NLAE-zl-P393
Bxylanisolvens_NLAE-zl-P352
Bxylanisolvens_NLAE-zl-P732
Bxylanisolvens_CL03T12C04
Bacteroides_1_1_30
Bacteroides_xylanisolvens_XB1A
Bacteroides_2_1_22
Bacteroides_D1
Bacteroides_ovatus_SD_CC_2a
Bacteroides_xylanisolvens_SD_CC_1b
Bacteroides_D22
Bxylanisolvens_NLAE-zl-C29
Bxylanisolvens_NLAE-zl-H194
Bxylanisolvens_NLAE-zl-G310
Bxylanisolvens_NLAE-zl-G421
Bxylanisolvens_NLAE-zl-C182
Bxylanisolvens_NLAE-zl-C339
Bacteroides_2_2_4
Bacteroides_D2
Bovatus_NLAE-zl-C11
Bovatus_NLAE-zl-C34
Bacteroides_ovatus_CL03T12C18
Bovatus_NLAE-zl-H59
Bovatus_NLAE-zl-H73
Bacteroides_ovatus_ATCC_8483
Bovatus_CL02T12C04
Bacteroides_ovatus_3_8_47FAA
Bacteroides_3_1_23
Bacteroides_ovatus_SD_CMC_3f
Bovatus_NLAE-zl-H304
Bovatus_NLAE-zl-H361

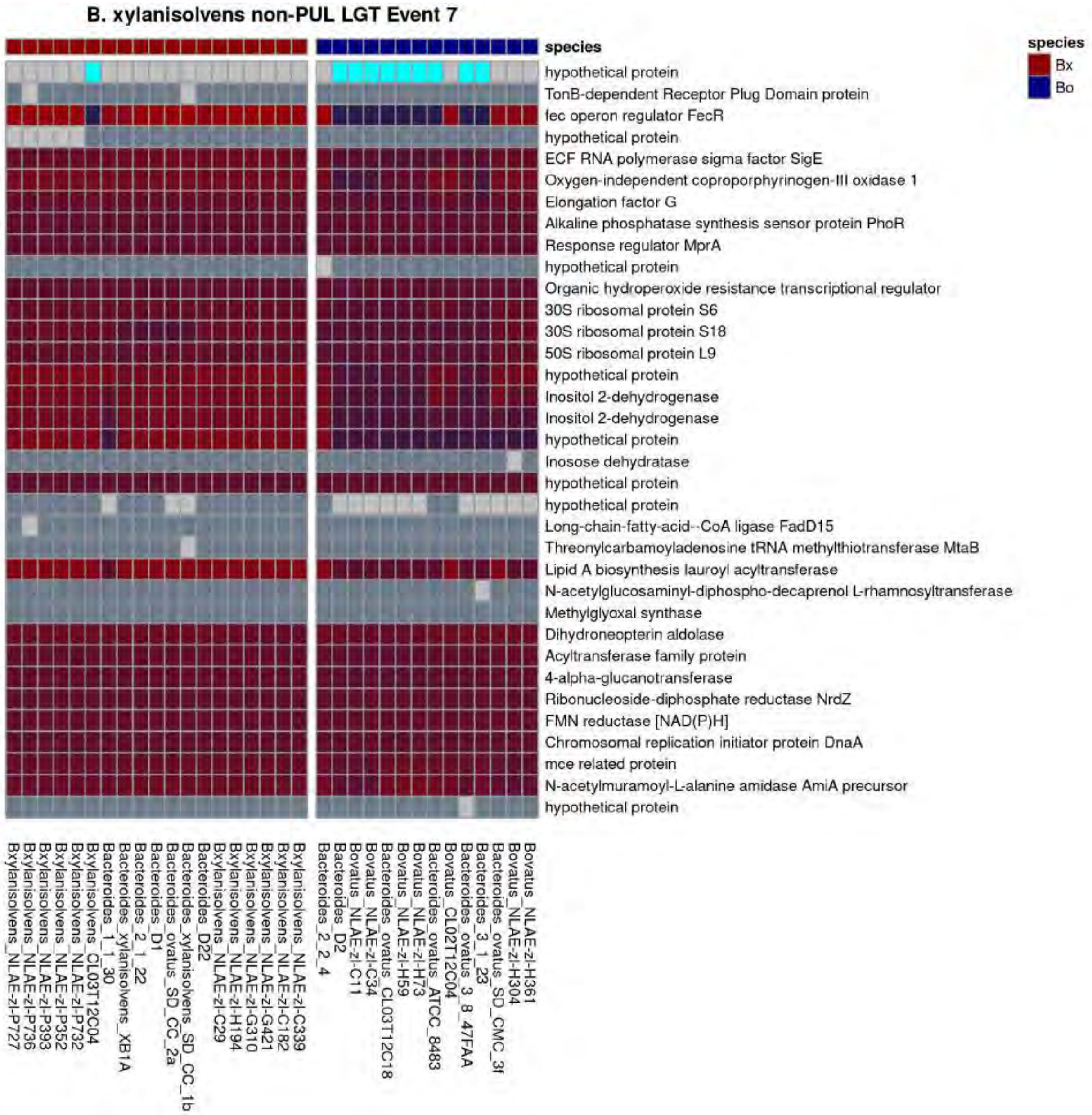
B. xylanisolvens non-PUL LGT Event 7
species
hypothetical protein
TonB-dependent Receptor Plug Domain protein
fec operon regulator FecR
hypothetical protein
ECF RNA polymerase sigma factor SigE
Oxygen-independent coproporphyrinogen-III oxidase 1
Elongation factor G
Alkaline phosphatase synthesis sensor protein PhoR
Response regulator MprA
hypothetical protein
Organic hydroperoxide resistance transcriptional regulator
30S ribosomal protein S6
30S ribosomal protein S18
50S ribosomal protein L9
hypothetical protein
Inositol 2-dehydrogenase
Inositol 2-dehydrogenase
hypothetical protein
Inosose dehydratase
hypothetical protein
hypothetical protein
Long-chain-fatty-acid--CoA ligase FadD15
Threonylcarbamoyladenosine tRNA methylthiotransferase MtaB
Lipid A biosynthesis lauroyl acyltransferase
N-acetylglucosaminyl-diphospho-decaprenol L-rhamnosyltransferase
Methylglyoxal synthase
Dihydroneopterin aldolase
Acyltransferase family protein
4-alpha-glucanotransferase
Ribonucleoside-diphosphate reductase NrdZ
FMN reductase [NAD(P)H]
Chromosomal replication initiator protein DnaA
mce related protein
N-acetylmuramoyl-L-alanine amidase AmiA precursor
hypothetical protein
species
Bx
Bo
Bxylanisolvens_NLAE-zl-P727
Bxylanisolvens_NLAE-zl-P736
Bxylanisolvens_NLAE-zl-P393
Bxylanisolvens_NLAE-zl-P352
Bxylanisolvens_NLAE-zl-P732
Bxylanisolvens_CL03T12C04
Bacteroides_1_1_30
Bacteroides_xylanisolvens_XB1A
Bacteroides_2_1_22
Bacteroides_D1
Bacteroides_ovatus_SD_CC_2a
Bacteroides_xylanisolvens_SD_CC_1b
Bacteroides_D22
Bxylanisolvens_NLAE-zl-C29
Bxylanisolvens_NLAE-zl-H194
Bxylanisolvens_NLAE-zl-G310
Bxylanisolvens_NLAE-zl-G421
Bxylanisolvens_NLAE-zl-C182
Bxylanisolvens_NLAE-zl-C339
Bacteroides_2_2_4
Bacteroides_D2
Bovatus_NLAE-zl-C11
Bovatus_NLAE-zl-C34
Bacteroides_ovatus_CL03T12C18
Bovatus_NLAE-zl-H59
Bovatus_NLAE-zl-H73
Bacteroides_ovatus_ATCC_8483
Bovatus_CL02T12C04
Bacteroides_ovatus_3_8_47FAA
Bacteroides_3_1_23
Bacteroides_ovatus_SD_CMC_3f
Bovatus_NLAE-zl-H304
Bovatus_NLAE-zl-H361

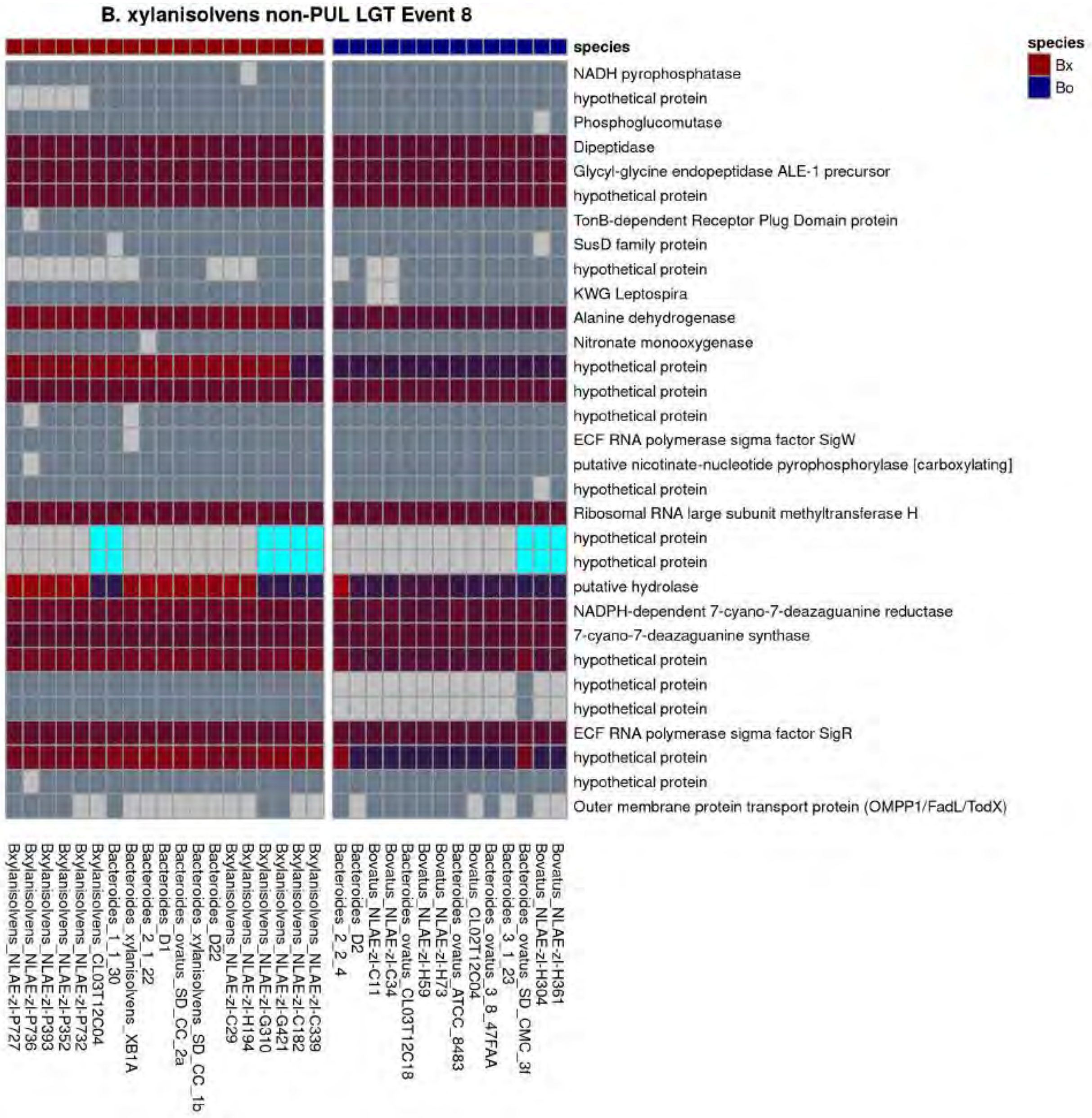

B. xylanisolvens non-PUL LGT Event 8
species
NADH pyrophosphatase
hypothetical protein
Phosphoglucomutase
Dipeptidase
Glycyl-glycine endopeptidase ALE-1 precursor
hypothetical protein
TonB-dependent Receptor Plug Domain protein
SusD family protein
hypothetical protein
KWG Leptospira
Alanine dehydrogenase
Nitronate monooxygenase
hypothetical protein
hypothetical protein
hypothetical protein
ECF RNA polymerase sigma factor SigW
putative nicotinate-nucleotide pyrophosphorylase [carboxylating]
hypothetical protein
Ribosomal RNA large subunit methyltransferase H
hypothetical protein
hypothetical protein
putative hydrolase
NADPH-dependent 7-cyano-7-deazaguanine reductase
7-cyano-7-deazaguanine synthase
hypothetical protein
hypothetical protein
hypothetical protein
ECF RNA polymerase sigma factor SigR
hypothetical protein
hypothetical protein
Outer membrane protein transport protein (OMPP1/FadL/TodX)
species
Bx
Bo
Bxylanisolvens_NLAE-zl-P727
Bxylanisolvens_NLAE-zl-P736
Bxylanisolvens_NLAE-zl-P393
Bxylanisolvens_NLAE-zl-P352
Bxylanisolvens_NLAE-zl-P732
Bxylanisolvens_CL03T12C04
Bacteroides_1_1_30
Bacteroides_xylanisolvens_XB1A
Bacteroides_2_1_22
Bacteroides_ovatus_SD_CC_2a
Bacteroides_D1
Bacteroides_xylanisolvens_SD_CC_1b
Bacteroides_D22
Bxylanisolvens_NLAE-zl-C29
Bxylanisolvens_NLAE-zl-H194
Bxylanisolvens_NLAE-zl-G310
Bxylanisolvens_NLAE-zl-G421
Bxylanisolvens_NLAE-zl-C182
Bxylanisolvens_NLAE-zl-C339
Bacteroides_2_2_4
Bacteroides_D2
Bovatus_NLAE-zl-C11
Bovatus_NLAE-zl-C34
Bacteroides_ovatus_CL03T12C18
Bovatus_NLAE-zl-H59
Bovatus_NLAE-zl-H73
Bacteroides_ovatus_ATCC_8483
Bovatus_CL02T12C04
Bacteroides_ovatus_3_8_47FAA
Bacteroides_3_1_23
Bacteroides_ovatus_SD_CMC_3f
Bovatus_NLAE-zl-H304
Bovatus_NLAE-zl-H361

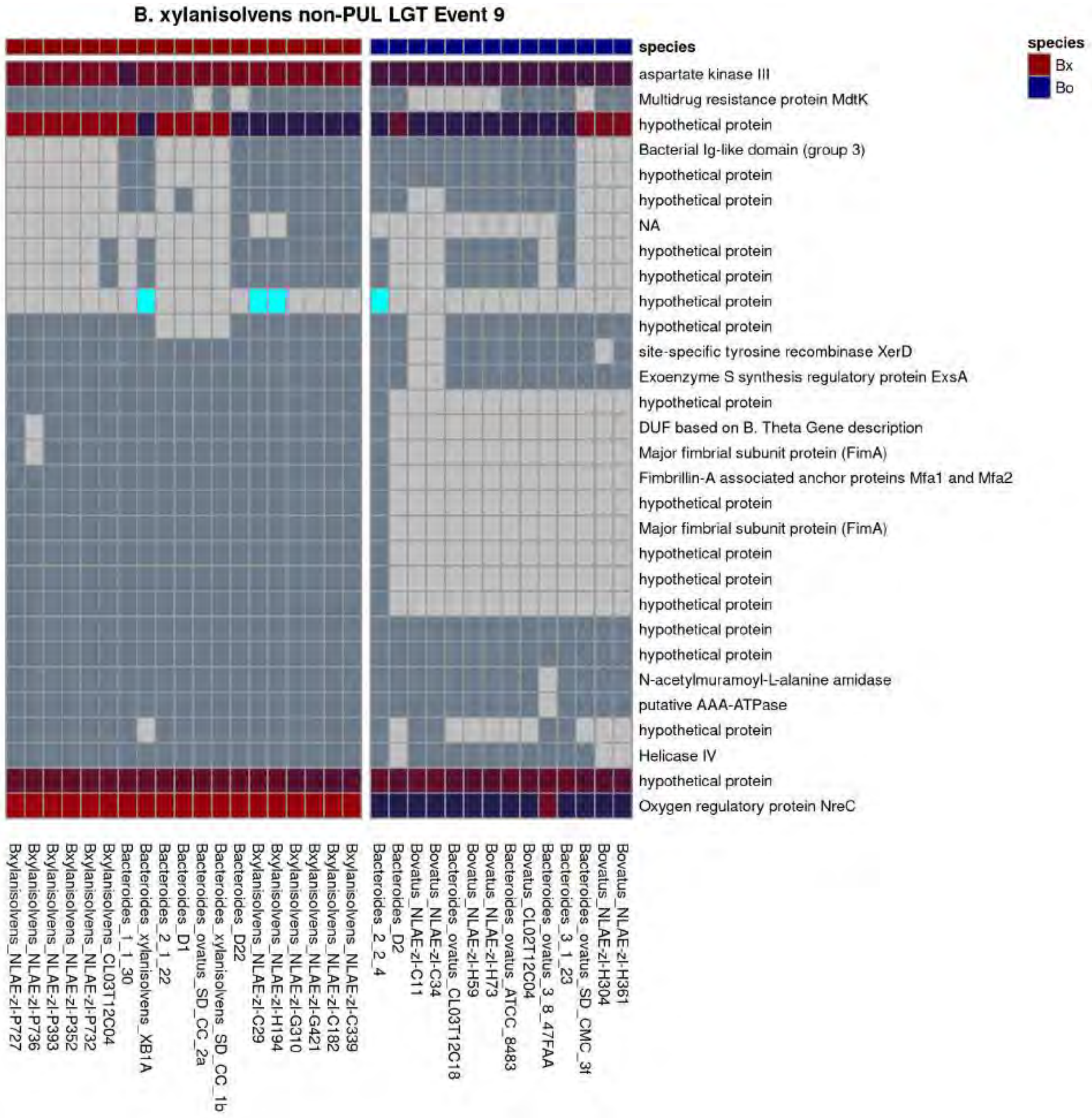
B. xylanisolvens non-PUL LGT Event 9
species
aspartate kinase III
Multidrug resistance protein MdtK
hypothetical protein
Bacterial Ig-like domain (group 3)
hypothetical protein
hypothetical protein
NA
hypothetical protein
hypothetical protein
hypothetical protein
hypothetical protein
site-specific tyrosine recombinase XerD
Exoenzyme S synthesis regulatory protein ExsA
hypothetical protein
DUF based on B. Theta Gene description
Major fimbrial subunit protein (FimA)
Fimbrillin-A associated anchor proteins Mfa1 and Mfa2
hypothetical protein
Major fimbrial subunit protein (FimA)
hypothetical protein
hypothetical protein
hypothetical protein
hypothetical protein
hypothetical protein
N-acetylmuramoyl-L-alanine amidase
putative AAA-ATPase
hypothetical protein
Helicase IV
hypothetical protein
Oxygen regulatory protein NreC
species
Bx
Bo
Bxylanisolvens_NLAE-zl-P727
Bxylanisolvens_NLAE-zl-P736
Bxylanisolvens_NLAE-zl-P393
Bxylanisolvens_NLAE-zl-P352
Bxylanisolvens_NLAE-zl-P732
Bxylanisolvens_CL03T12C04
Bacteroides_1_1_30
Bacteroides_xylanisolvens_XB1A
Bacteroides_2_1_22
Bacteroides_D1
Bacteroides_ovatus_SD_CC_2a
Bacteroides_xylanisolvens_SD_CC_1b
Bacteroides_D22
Bxylanisolvens_NLAE-zl-C29
Bxylanisolvens_NLAE-zl-H194
Bxylanisolvens_NLAE-zl-G310
Bxylanisolvens_NLAE-zl-G421
Bxylanisolvens_NLAE-zl-C182
Bxylanisolvens_NLAE-zl-C339
Bacteroides_2_2_4
Bacteroides_D2
Bovatus_NLAE-zl-C11
Bovatus_NLAE-zl-C34
Bacteroides_ovatus_CL03T12C18
Bovatus_NLAE-zl-H59
Bovatus_NLAE-zl-H73
Bacteroides_ovatus_ATCC_8483
Bovatus_CL02T12C04
Bacteroides_ovatus_3_8_47FAA
Bacteroides_3_1_23
Bacteroides_ovatus_SD_CMC_3f
Bovatus_NLAE-zl-H304
Bovatus_NLAE-zl-H361

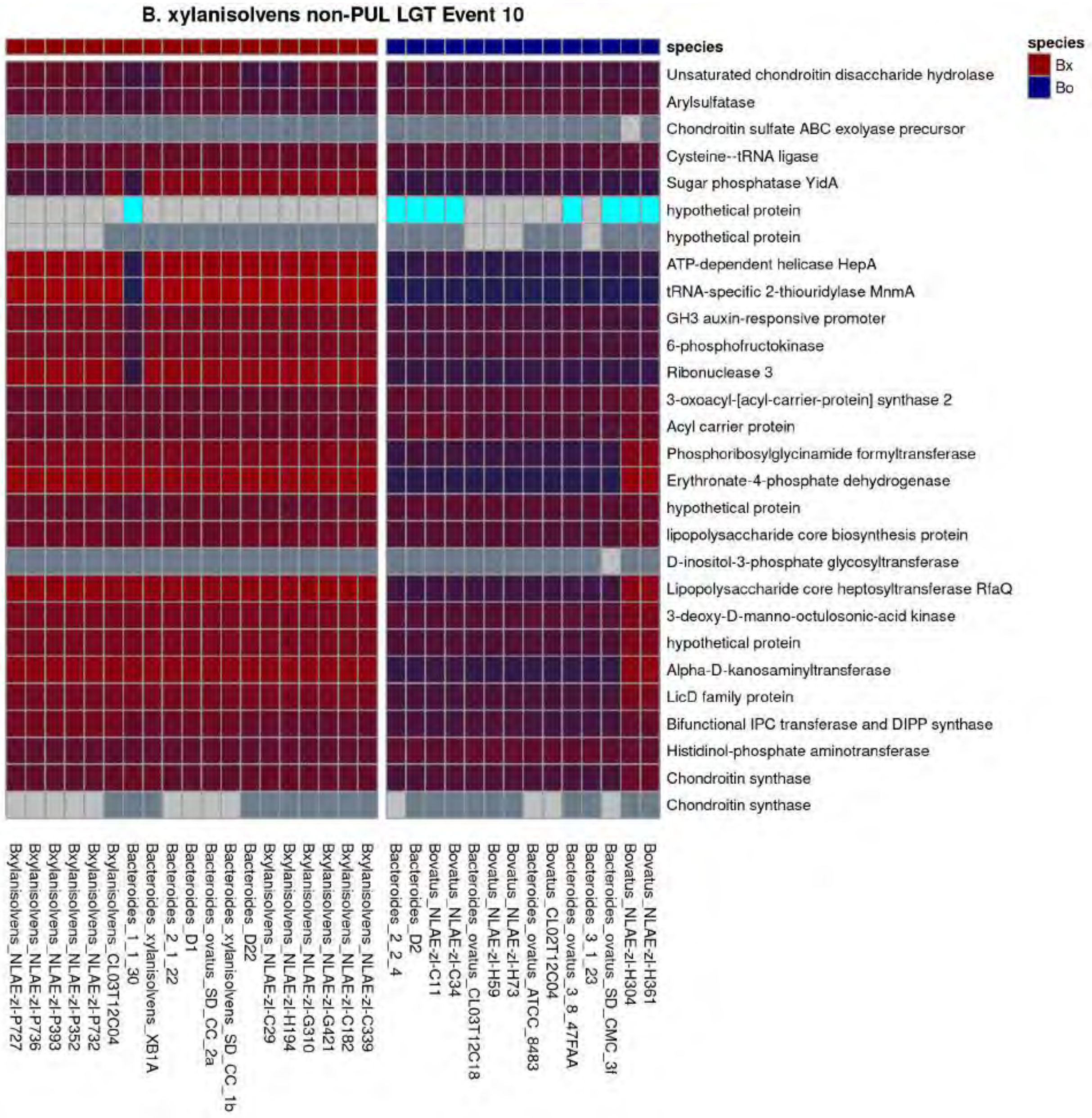
B. xylanisolvens non-PUL LGT Event 10
species
Bx
Bo
Unsaturated chondroitin disaccharide hydrolase
Arylsulfatase
Chondroitin sulfate ABC exolyase precursor
Cysteine--tRNA ligase
Sugar phosphatase YidA
hypothetical protein
hypothetical protein
ATP-dependent helicase HepA
tRNA-specific 2-thiouridylase MnmA
GH3 auxin-responsive promoter
6-phosphofructokinase
Ribonuclease 3
3-oxoacyl-[acyl-carrier-protein] synthase 2
Acyl carrier protein
Phosphoribosylglycinamide formyltransferase
Erythronate-4-phosphate dehydrogenase
hypothetical protein
lipopolysaccharide core biosynthesis protein
D-inositol-3-phosphate glycosyltransferase
Lipopolysaccharide core heptosyltransferase RfaQ
3-deoxy-D-manno-octulosonic-acid kinase
hypothetical protein
Alpha-D-kanosaminyltransferase
LicD family protein
Bifunctional IPC transferase and DIPP synthase
Histidinol-phosphate aminotransferase
Chondroitin synthase
Chondroitin synthase
Bxylanisolvens_NLAE-zl-P727
Bxylanisolvens_NLAE-zl-P736
Bxylanisolvens_NLAE-zl-P393
Bxylanisolvens_NLAE-zl-P352
Bxylanisolvens_NLAE-zl-P732
Bxylanisolvens_CL03T12C04
Bacteroides_1_1_30
Bacteroides_xylanisolvens_XB1A
Bacteroides_2_1_22
Bacteroides_D1
Bacteroides_ovatus_SD_CC_2a
Bacteroides_xylanisolvens_SD_CC_1b
Bacteroides_D22
Bxylanisolvens_NLAE-zl-C29
Bxylanisolvens_NLAE-zl-H194
Bxylanisolvens_NLAE-zl-G310
Bxylanisolvens_NLAE-zl-G421
Bxylanisolvens_NLAE-zl-C182
Bxylanisolvens_NLAE-zl-C339
Bacteroides_2_2_4
Bacteroides_D2
Bovatus_NLAE-zl-C11
Bovatus_NLAE-zl-C34
Bacteroides_ovatus_CL03T12C18
Bovatus_NLAE-zl-H59
Bovatus_NLAE-zl-H73
Bacteroides_ovatus_ATCC_8483
Bovatus_CL02T12C04
Bacteroides_ovatus_3_8_47FAA
Bacteroides_3_1_23
Bacteroides_ovatus_SD_CMC_3f
Bovatus_NLAE-zl-H304
Bovatus_NLAE-zl-H361

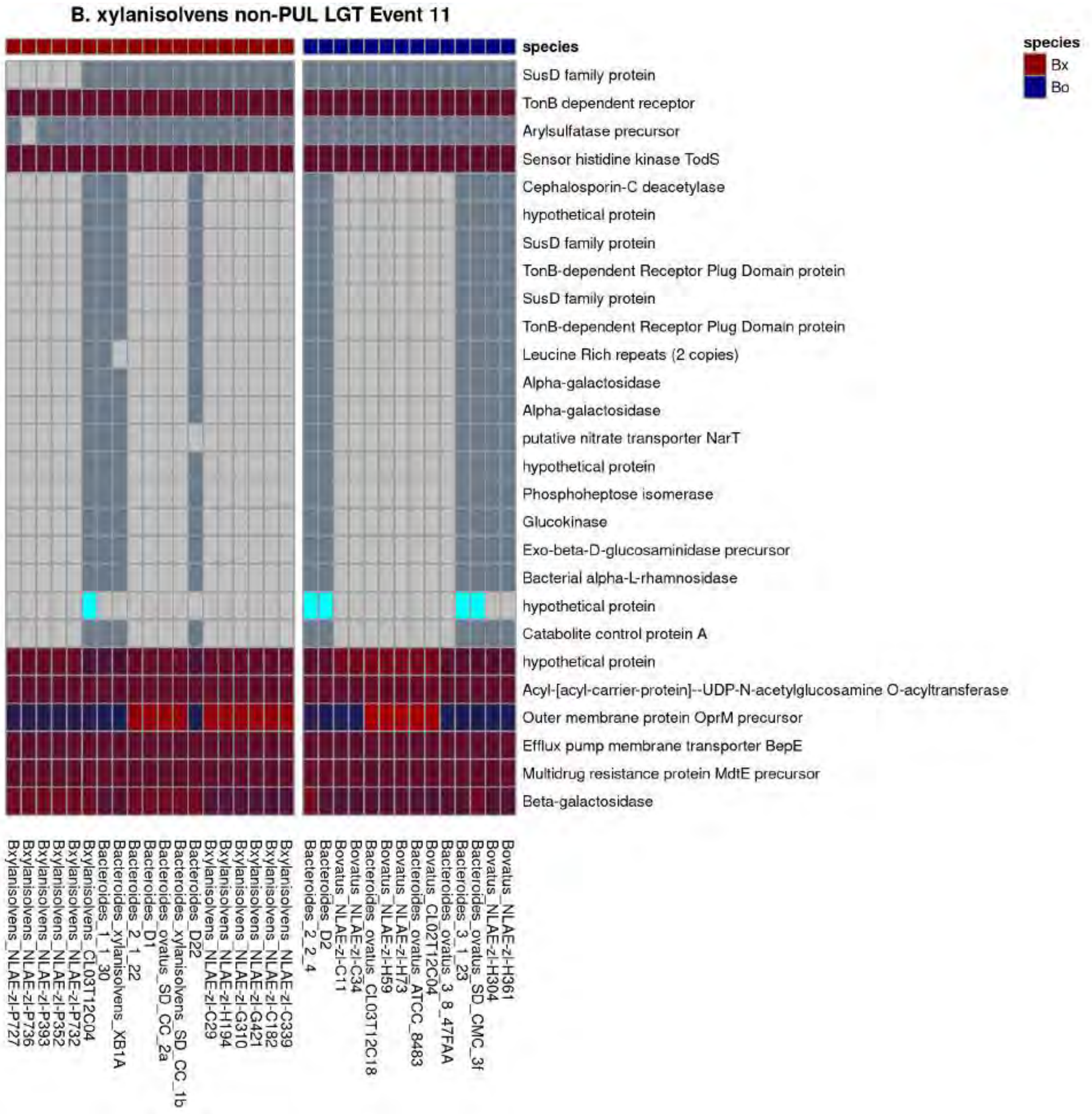

B. xylanisolvens non-PUL LGT Event 11
species
SusD family protein
TonB dependent receptor
Arylsulfatase precursor
Sensor histidine kinase TodS
Cephalosporin-C deacetylase
hypothetical protein
SusD family protein
TonB-dependent Receptor Plug Domain protein
SusD family protein
TonB-dependent Receptor Plug Domain protein
Leucine Rich repeats (2 copies)
Alpha-galactosidase
Alpha-galactosidase
putative nitrate transporter NarT
hypothetical protein
Phosphoheptose isomerase
Glucokinase
Exo-beta-D-glucosaminidase precursor
Bacterial alpha-L-rhamnosidase
hypothetical protein
Catabolite control protein A
hypothetical protein
Acyl-[acyl-carrier-protein]--UDP-N-acetylglucosamine O-acyltransferase
Outer membrane protein OprM precursor
Efflux pump membrane transporter BepE
Multidrug resistance protein MdtE precursor
Beta-galactosidase
species
Bx
Bo
Bxylanisolvens_NLAE-zl-P727
Bxylanisolvens_NLAE-zl-P736
Bxylanisolvens_NLAE-zl-P393
Bxylanisolvens_NLAE-zl-P352
Bxylanisolvens_NLAE-zl-P732
Bxylanisolvens_CL03T12C04
Bacteroides_1_1_30
Bacteroides_xylanisolvens_XB1A
Bacteroides_2_1_22
Bacteroides_D1
Bacteroides_ovatus_SD_CC_2a
Bacteroides_xylanisolvens_SD_CC_1b
Bacteroides_D22
Bxylanisolvens_NLAE-zl-C29
Bxylanisolvens_NLAE-zl-H194
Bxylanisolvens_NLAE-zl-G310
Bxylanisolvens_NLAE-zl-G421
Bxylanisolvens_NLAE-zl-C182
Bxylanisolvens_NLAE-zl-C339
Bacteroides_2_2_4
Bacteroides_D2
Bovatus_NLAE-zl-C11
Bovatus_NLAE-zl-C34
Bacteroides_ovatus_CL03T12C18
Bovatus_NLAE-zl-H59
Bovatus_NLAE-zl-H73
Bacteroides_ovatus_ATCC_8483
Bovatus_CL02T12C04
Bacteroides_ovatus_3_8_47FAA
Bacteroides_3_1_23
Bacteroides_ovatus_SD_CMC_3f
Bovatus_NLAE-zl-H304
Bovatus_NLAE-zl-H361

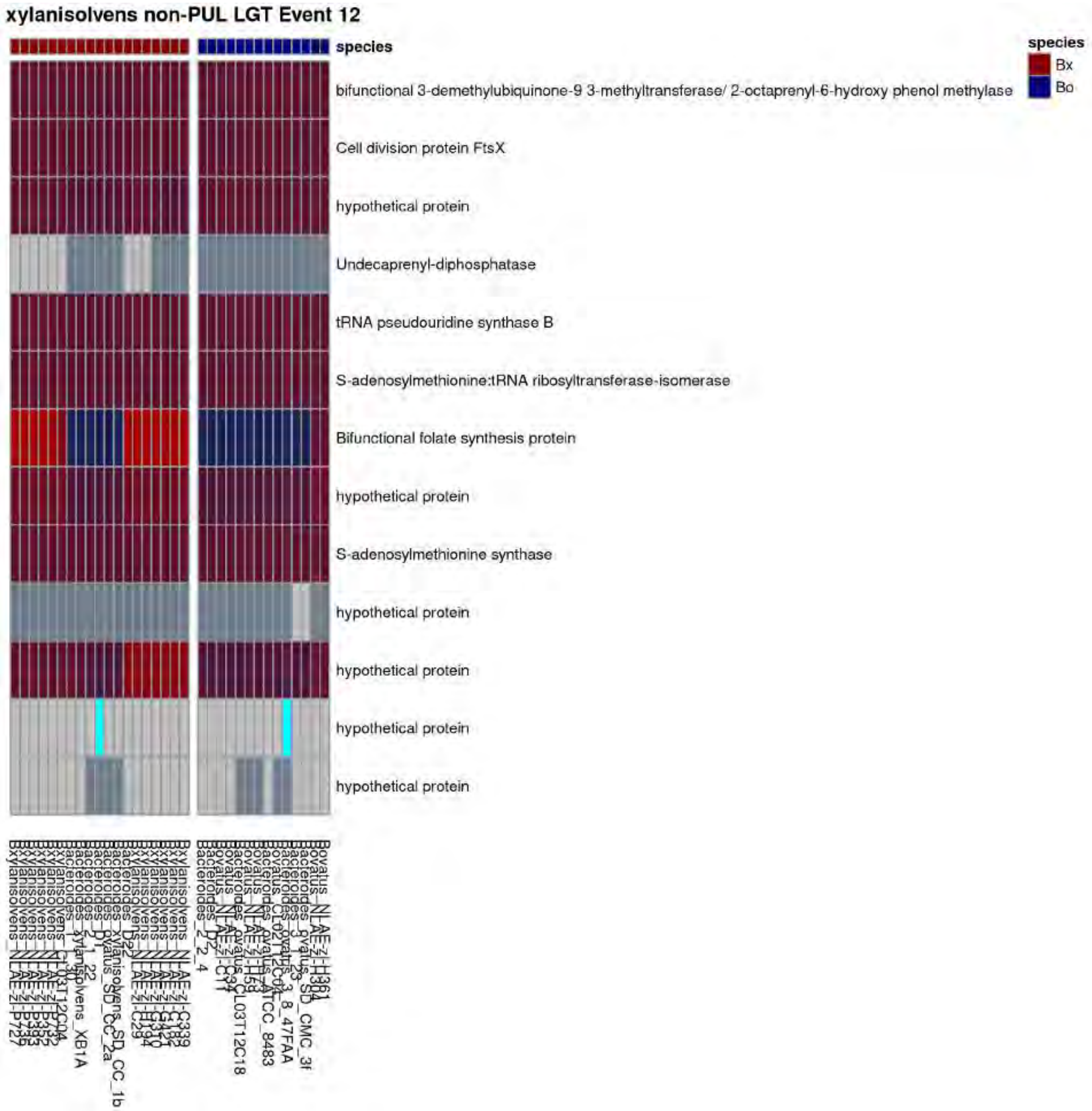
xylanisolvens non-PUL LGT Event 12
species
bifunctional 3-demethylubiquinone-9 3-methyltransferase/ 2-octaprenyl-6-hydroxy phenol methylase
Cell division protein FtsX
hypothetical protein
Undecaprenyl-diphosphatase
tRNA pseudouridine synthase B
S-adenosylmethionine:tRNA ribosyltransferase-isomerase
Bifunctional folate synthesis protein
hypothetical protein
S-adenosylmethionine synthase
hypothetical protein
hypothetical protein
hypothetical protein
hypothetical protein
species
Bx
Bo

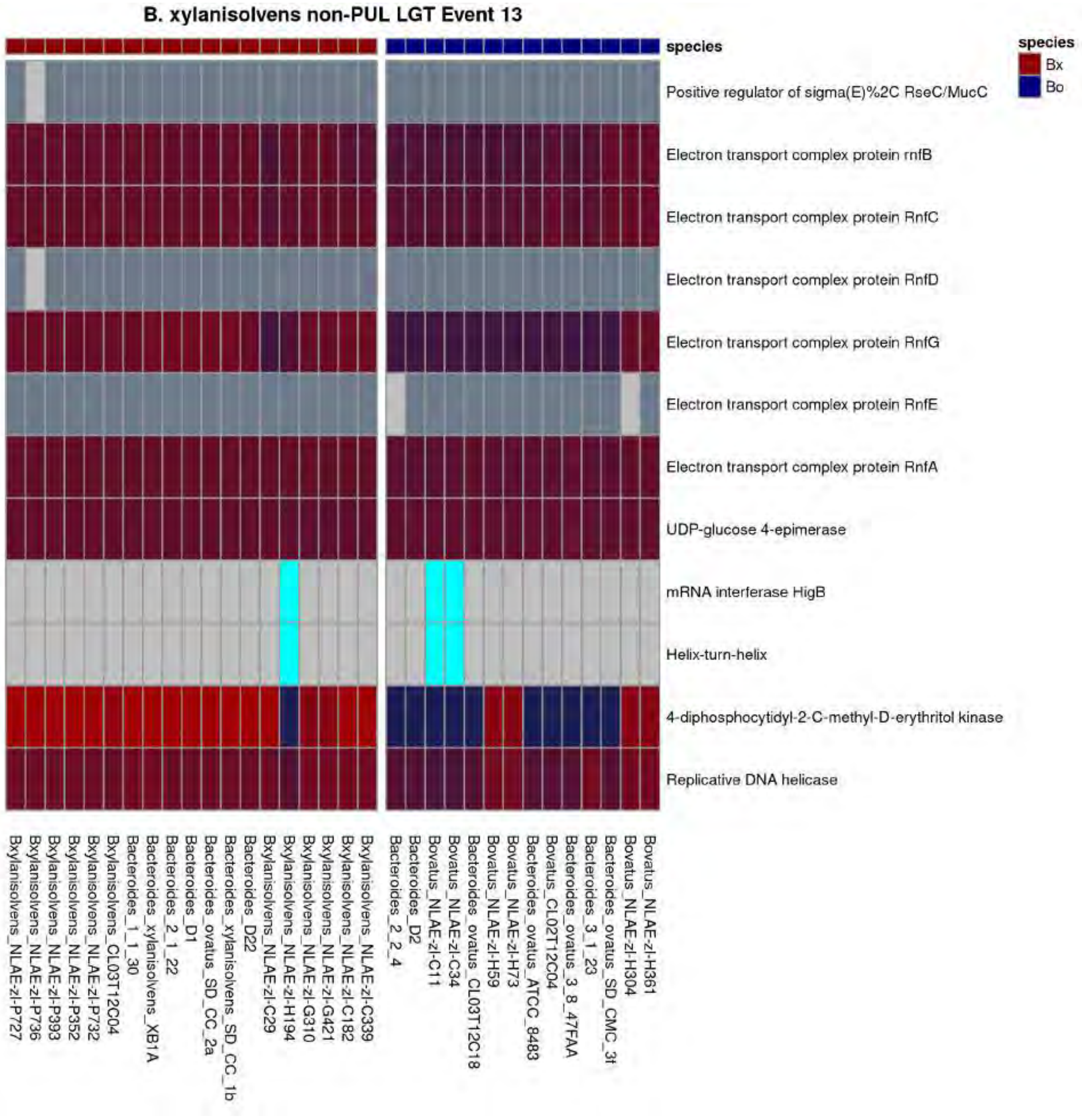
B. xylanisolvens non-PUL LGT Event 13
species
Bx
Bo
Positive regulator of sigma(E)%2C RseC/MucC
Electron transport complex protein rnfB
Electron transport complex protein RnfC
Electron transport complex protein RnfD
Electron transport complex protein RnfG
Electron transport complex protein RnfE
Electron transport complex protein RnfA
UDP-glucose 4-epimerase
mRNA interferase HigB
Helix-turn-helix
4-diphosphocytidyl-2-C-methyl-D-erythritol kinase
Replicative DNA helicase
Bxylanisolvens_NLAE-zl-P727
Bxylanisolvens_NLAE-zl-P736
Bxylanisolvens_NLAE-zl-P393
Bxylanisolvens_NLAE-zl-P352
Bxylanisolvens_NLAE-zl-P732
Bxylanisolvens_CL03T12C04
Bacteroides_1_1_30
Bacteroides_xylanisolvens_XB1A
Bacteroides_2_1_22
Bacteroides_D1
Bacteroides_ovatus_SD_CC_2a
Bacteroides_xylanisolvens_SD_CC_1b
Bacteroides_D22
Bxylanisolvens_NLAE-zl-C29
Bxylanisolvens_NLAE-zl-H194
Bxylanisolvens_NLAE-zl-G310
Bxylanisolvens_NLAE-zl-G421
Bxylanisolvens_NLAE-zl-C182
Bxylanisolvens_NLAE-zl-C339
Bacteroides_2_2_4
Bacteroides_D2
Bovatus_NLAE-zl-C11
Bovatus_NLAE-zl-C34
Bacteroides_ovatus_CL03T12C18
Bovatus_NLAE-zl-H59
Bovatus_NLAE-zl-H73
Bacteroides_ovatus_ATCC_8483
Bovatus_CL02T12C04
Bacteroides_ovatus_3_8_47FAA
Bacteroides_3_1_23
Bacteroides_ovatus_SD_CMC_3f
Bovatus_NLAE-zl-H304
Bovatus_NLAE-zl-H361
